# Strigolactones positively regulate abscisic acid-dependent heat and cold tolerance in tomato

**DOI:** 10.1038/s41438-021-00668-y

**Published:** 2021-11-01

**Authors:** Cheng Chi, Xuechen Xu, Mengqi Wang, Hui Zhang, Pingping Fang, Jie Zhou, Xiaojian Xia, Kai Shi, Yanhong Zhou, Jingquan Yu

**Affiliations:** 1 Department of Horticulture, Zijingang Campus, Zhejiang University, 866 Yuhangtang Road, Hangzhou, 310058 P.R. China; 2 Zhejiang Provincial Key Laboratory of Horticultural Plant Integrative Biology, 866 Yuhangtang Road, Hangzhou, 310058 P.R. China; 3 Key Laboratory of Horticultural Plants Growth, Development and Quality Improvement, Agricultural Ministry of China, 866 Yuhangtang Road, Hangzhou, 310058 P.R. China

**Keywords:** Metabolism, Plant sciences, Systems biology

## Abstract

Strigolactones are carotenoid-derived phytohormones that impact plant growth and development in diverse ways. However, the roles of strigolactones in the responses to temperature stresses are largely unknown. Here, we demonstrated that strigolactone biosynthesis is induced in tomato (*Solanum lycopersicum*) by heat and cold stresses. Compromised strigolactone biosynthesis or signaling negatively affected heat and cold tolerance, while application of the synthetic strigolactone analog GR24^5DS^ enhanced heat and cold tolerance. Strigolactone-mediated heat and cold tolerance was associated with the induction of abscisic acid (ABA), heat shock protein 70 (HSP70) accumulation, *C-REPEAT BINDING FACTOR 1* (*CBF1*) transcription, and antioxidant enzyme activity. Importantly, a deficiency in ABA biosynthesis compromised the GR24^5DS^ effects on heat and cold stresses and abolished the GR24^5DS^-induced transcription of *HSP70*, *CBF1*, and antioxidant-related genes. These results support that strigolactones positively regulate tomato heat and cold tolerance and that they do so at least partially by the induction of CBFs and HSPs and the antioxidant response in an ABA-dependent manner.

## Introduction

Plants encounter stressful conditions that adversely impact growth, metabolism, and productivity throughout their life cycles. Extreme high or low temperature, drought, salinity, floods, pollutants, and radiation are the main stress factors that limit the productivity of many crops of economic importance^1^. These stresses disrupt many physiological processes through the excessive generation of reactive oxygen species (ROS), which results in serious injury to DNA and proteins in plants^2,3^. To avoid oxidative damage, plants activate ROS-scavenging enzymes, including glutathione reductase (GR), superoxide dismutase (SOD) and ascorbate peroxidase (APX), and make use of nonenzymatic antioxidants, including glutathione and ascorbate^3,4^. The induction of the antioxidant system is considered a crucial mechanism to enhance extreme temperature tolerance in plants, as transcript suppression or deficiency of the genes encoding antioxidants results in increased sensitivity to temperature stresses^5,6^.

Plants have evolved a complex network of interconnected signaling pathways allowing them to flexibly acclimate to and overcome these stress conditions. In addition to the induction of the antioxidant system, plants have also evolved other mechanisms to prevent cellular damage in response to temperature stresses^7,8^. Heat shock proteins (HSPs) are significantly induced by heat stress to protect cellular proteins against irreversible damage^9^. HSP70s comprise a subset of HSPs and function as molecular chaperones to bind and release unfolded/nonnative proteins. Several studies have shown that HSP70s are pivotal for the survival of plants under heat stress conditions and the induction of thermotolerance^9–11^. In contrast, plants can also activate the transcription of *C-REPEAT BINDING FACTOR* (*CBF*), subsequently leading to the induction of many *COLD-RESPONSIVE* (*COR*) genes modulating cold stress responses^7,12^.

Plant hormones such as abscisic acid (ABA) are actively involved in stress responses^13^. In response to stresses, plants accumulate more ABA in leaves, which can promote stomatal closure, enhance water balance, and induce antioxidant defense systems to alleviate oxidative injury^13,14^. Moreover, ABA can activate numerous cellular responses in plants through a series of signal transduction pathways and induction of *HSP* and *CBF*, promoting plant tolerance against stresses^11,15^. Furthermore, ABA can interact with other plant hormones, such as gibberellins, jasmonic acid and strigolactones, which makes it a hub in the responses to various abiotic stresses^7,16,17^.

Strigolactones are a group of terpenoid lactone hormones. To date, several genes involved in the biosynthesis of strigolactones, such as *CAROTENOID CLEAVAGE DIOXYGENASE 7* (*CCD7*), *CCD8*, and *MORE AXILLARY GROWTH 1* (*MAX1*), have been identified^18–20^. In Arabidopsis, for example, after perception of the strigolactone signal via the strigolactone-specific receptor DWARF14 (D14), downstream proteins such as the F-box protein MAX2 participate in various physiological processes^21,22^. Deficiency or suppressed transcription of strigolactone biosynthesis or signaling genes has been shown to affect multiple processes in plants, such as leaf senescence, root growth, shoot branching, and responses to external stimuli, including pathogens, nutrient deficiency, drought, and salinity^23–28^. Strigolactone biosynthesis is induced under adverse growth conditions such as phosphate deficiency^29^. Strigolactone-deficient and/or strigolactone-insensitive mutants are hypersensitive to stresses such as salt, drought and osmotic stress^26,30,31^, and exogenous strigolactone treatment rescues the phenotype of wild-type plants and strigolactone-deficient *max* mutants (*max3-11*, *max4-7*) under drought stress in Arabidopsis^26^, implying that strigolactones positively regulate abiotic stress tolerance. Notably, decreased stress tolerance in strigolactone-related mutants is associated with lower ABA levels and/or slower ABA-mediated stomatal closure in stressed shoots of several species, while exogenous strigolactones increase the sensitivity of guard cells to ABA in tomato, *Lotus japonicus*, and Arabidopsis^26,30–32^. Therefore, the crosstalk between strigolactones and ABA may play a critical role in the plant response to stresses.

Strigolactones are mainly produced in roots but can be transported to shoots or secreted into the rhizosphere^33,34^. To date, several studies have shown that strigolactone biosynthesis or signaling is important for plants to acclimate to temperature stresses. Cooper et al.^35^ demonstrated that there is a more significant decrease in the CO_2_ assimilation rate in several strigolactone biosynthesis or signaling mutants of pea and Arabidopsis after a dark chill. Similarly, Hu et al.^36^ found that application of strigolactones attenuates heat suppression at leaf elongation, which is associated with the increased transcript levels of cell cycle-related genes and decreased transcript levels of genes involved in auxin transport in elongating leaves of tall fescue. Most recently, Liu et al.^17^ showed that rice *dwarf27* mutants deficient in strigolactone biosynthesis display lower ABA contents with decreased transcript levels of ABA-responsive genes and impaired cold resistance. In the present work, we report that strigolactone biosynthesis and signaling play crucial roles in the responses to heat and cold stresses in tomato. The results show that strigolactones act as positive regulators of tolerance to heat and cold stresses by activating the transcription of *CBF* and *HSP* and antioxidant enzyme activity at least partially in an ABA-dependent manner.

## Results

### Strigolactones accumulate in the roots in response to heat and cold stresses

**Fig. 1:**
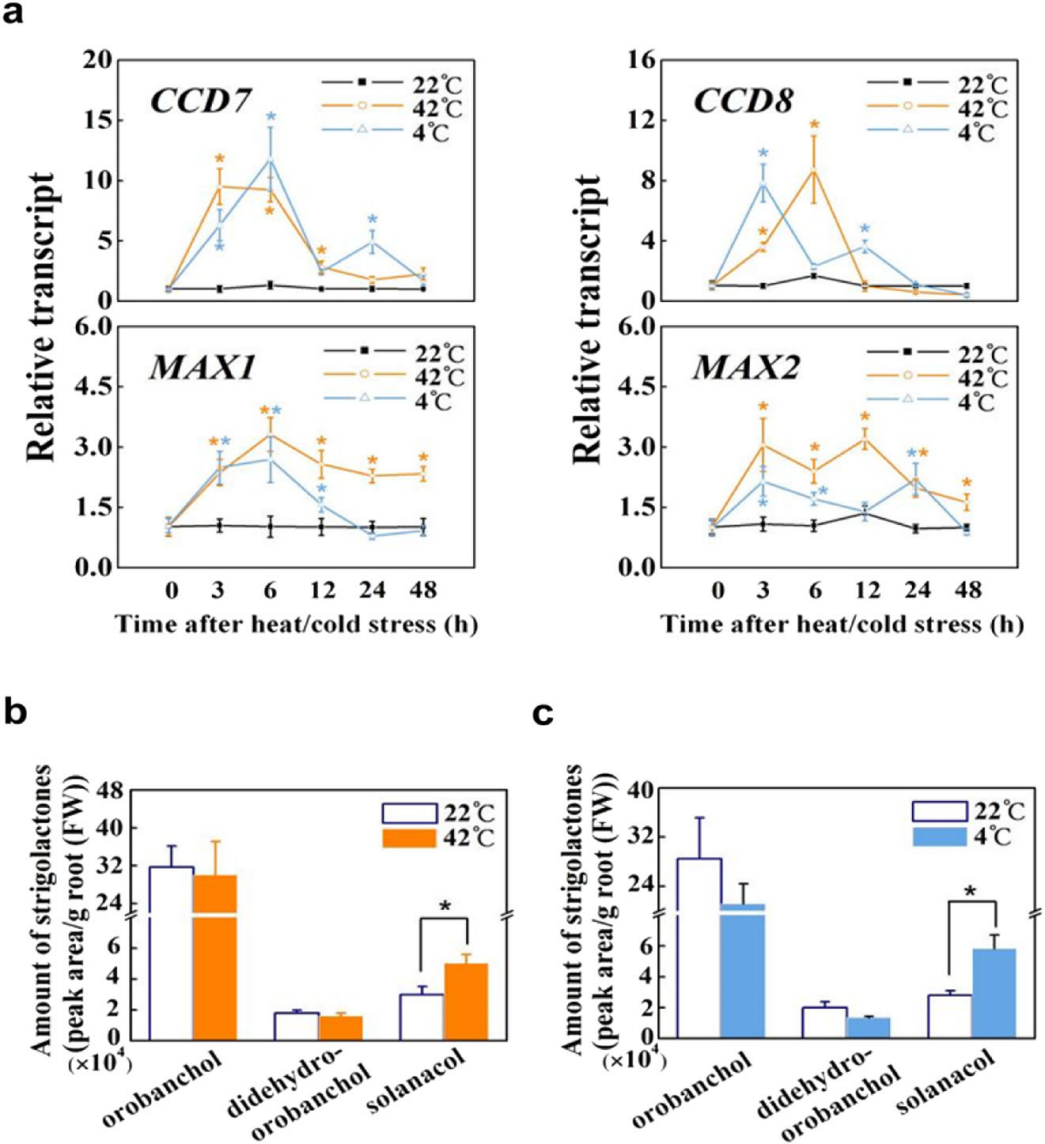
Strigolactone biosynthesis is induced by heat and cold stresses in tomato. **a** Time course of the transcript levels of strigolactone biosynthesis (*CCD7*, *CCD8*, and *MAX1*) and signaling (*MAX2*) genes at 42 or 4 °C. RNA was extracted from the root samples of wild-type (WT; Ailsa Craig) plants collected during the first 48 h of heat and cold stresses. **b**, **c** Amounts of strigolactones in WT roots under heat (**b**) or cold (**c**) stress. Root samples were collected after 3 h at 42 °C and after 6 h at 4 °C. The results are mean ± SD of three biological replicates. Student’s *t*-test was adopted at the *P* < 0.05 level, and significant differences are indicated by asterisks. For (**a**), asterisks indicate significant differences compared with the plants at 22 °C at each indicated time point

To explore the role of strigolactones under extreme temperatures, a time course of the transcript levels of strigolactone biosynthesis (*CCD7*, *CCD8*, and *MAX1*) and signaling (*MAX2*) genes in the roots of wild-type tomato plants was carried out after the whole plants were transferred to hot (42 °C) or cold (4 °C) conditions. As shown in Fig. 1a, either high or low growth temperature significantly induced the transcription of *CCD7*, *CCD8*, *MAX1*, and *MAX2* in the roots at 3 h after heat or at 6 h after cold stress. Afterward, the transcript levels of these genes decreased gradually to levels similar to those before the stresses. Among the four genes examined, the increases in the transcript levels of *CCD7* and *CCD8* were more notable than those of *MAX1* and *MAX2* under either high- or low-temperature conditions. Additional experiments showed that transcript levels of strigolactone biosynthesis genes in the leaves were also induced in response to heat or cold stress, and the levels were, however, much lower than those in the roots under optimal growth conditions, and hot and cold conditions (Figs. 1a and S1). In addition, changes in the accumulation of three principal strigolactones, orobanchol, solanacol, and didehydro-orobanchol, in the roots were examined (Fig. 1b, c). While the levels of orobanchol and didehydro-orobanchol were not significantly altered, solanacol significantly increased under either high- or low-temperature conditions by 68.7% at 3 h after heat stress and by 107.8% at 6 h after cold stress (Fig. 1b, c). These results indicate that strigolactone biosynthesis is induced at the early stage of heat or cold stress.

### Strigolactones play a positive role in the heat stress response and HSP70 protein accumulation under heat stress

To examine whether strigolactones regulate heat responses, virus-induced gene silencing (VIGS) experiments were conducted, and we generated *CCD7*-, *CCD8*-, *MAX1*-, and *MAX2*-silenced plants with a reduction in the respective gene transcript levels in the roots by 79.8%, 77.4%, 76.2%, and 79.9% as well as a reduction in the gene transcript levels of *CCD7*, *CCD8*, and *MAX1* in the leaves by 74.1%, 73.7%, and 71.7% in *CCD7*-, *CCD8*-, and *MAX1*-silenced plants, respectively, compared with those in the control plants (pTRV) (Fig. S2a, b). In addition to the decreasing effects on stimulating the germination of *Phelipanche aegyptiaca* seeds, the contents of orobanchol, didehydro-orobanchol, and solanacol in root extracts of the *CCD7*-, *CCD8*-, and *MAX1*-silenced plants was significantly lower than that of the pTRV plants (Fig. S2c, d). However, root extracts of *MAX2*-silenced plants did not show altered accumulation of these strigolactones or result in changed germination of *P.* *aegyptiaca* seeds (Fig. S2c, d). Therefore, strigolactone biosynthesis was partially suppressed in *CCD7*-, *CCD8*-, and *MAX1*-silenced plants but not in *MAX2*-silenced plants. Moreover, all the VIGS plants at the 6~7-leaf stage were shorter (Fig. S3a) and had increased root weight with little change in shoot weight (Fig. S3c) compared with the pTRV plants. Moreover, increased lateral branches were found in all VIGS plants at the 10~12-leaf stage (Fig. S3b). Additionally, detached leaves from the VIGS plants exhibited increased sensitivity to dehydration, as indicated by the increased water loss under dehydration conditions and higher stomatal conductance than that in the pTRV plants (Fig. S4). Interestingly, higher leaf water loss rates during dehydration and increased stomatal conductance were found in *MAX2*-silenced plants than those in *CCD7-*, *CCD8-*, and *MAX1*-silenced plants (Fig. S4b, c). Therefore, suppression of either strigolactone biosynthesis or signaling was sufficient to alter plant architecture and water dynamics in tomato plants.

**Fig. 2:**
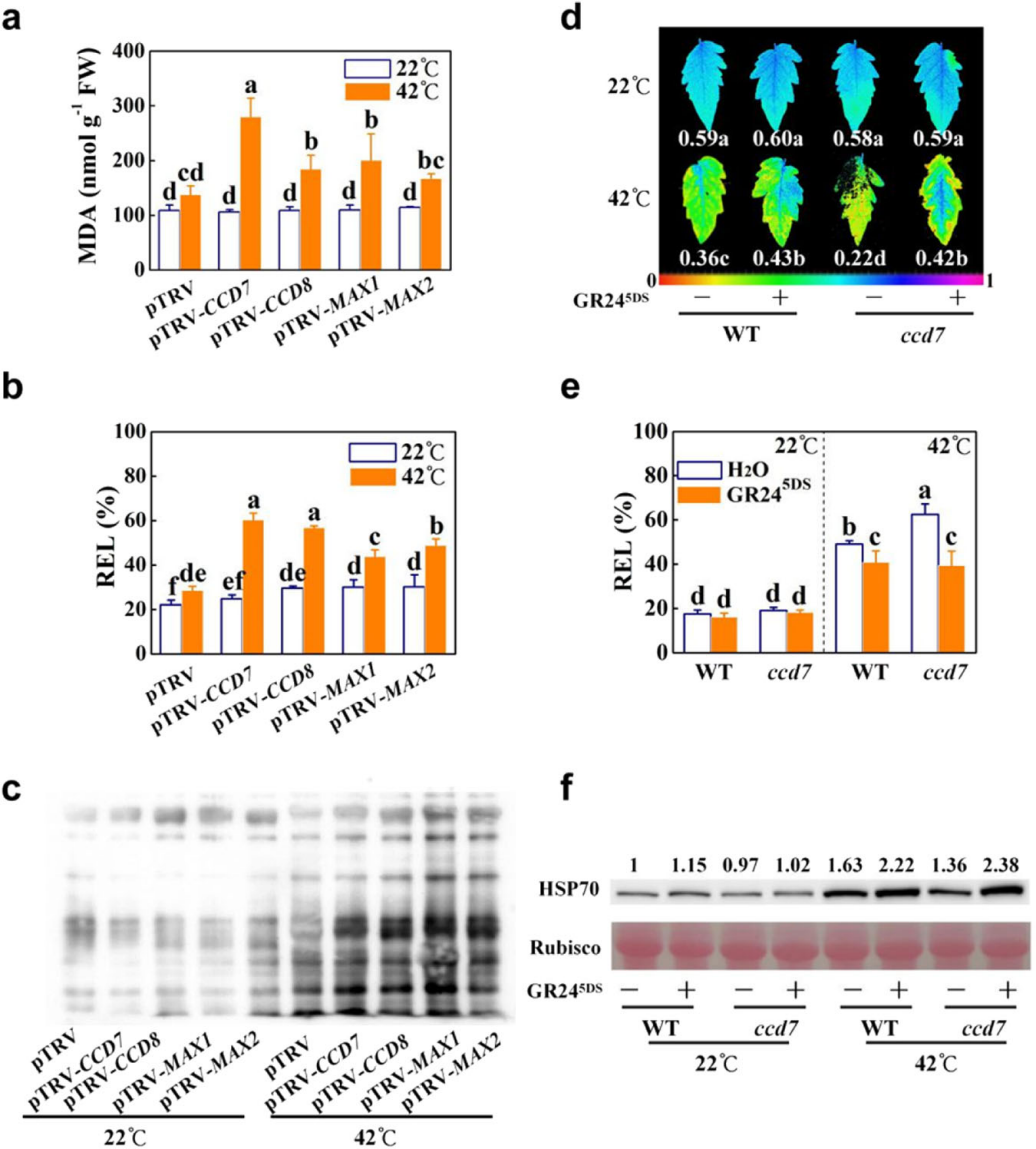
Effects of suppression of strigolactone biosynthesis and signaling and of GR24^5DS^ application on the heat response in tomato. **a** Malondialdehyde (MDA) accumulation in the leaves after 48 h of heat at 42 °C. **b**, **e** Relative electrolyte leakage (REL) in the leaves after 48 h of heat at 42 °C. **c** Oxidized proteins in the leaves after 12 h of heat at 42 °C. **d** Actual quantum efficiency of PSII photochemistry (Ф_PSII_) of the leaves after 48 h of heat at 42 °C. The color scale below the image ranges from 0 to 1.0 (purple). **f** HSP70 protein accumulation in the leaves after 12 h of heat at 42 °C. The number above each lane represents the relative band intensity value. For (**a**–**c**), the plants used for gene silencing were the Ailsa Craig background. Plants transformed with the empty vector pTRV served as controls. For (**d**–**f**), WT refers to the wild type (Condine Red), and *ccd7* refers to transgenic CRISPR-*ccd7* mutants. The GR24^5DS^ (3 µM, 15 mL) solution was applied to the roots of each plant 24 h before heat stress at 42 °C. The plus and minus marks represent the application of GR24^5DS^ and water solution, respectively. The results in (**a**, **b**, **e**) are mean ± SD of three biological replicates. For (**d**), 15 leaves were used. Significant differences are indicated by different letters (*P* < 0.05, Tukey’s test)

After 48 h of heat stress at 42 °C, the pTRV plants exhibited slight dehydration symptoms (Fig. S5). In comparison, leaves on the VIGS plants showed severe wilting after the same heat treatment (Fig. S5). Moreover, higher malondialdehyde (MDA) contents were observed in these VIGS plants than in the pTRV plants (Fig. 2a). Relative electrolyte leakage (REL) increased slightly in the leaves of the VIGS plants at 22 °C (Fig. 2b). Heat stress caused a more significant increase in the REL values in VIGS plants, and this increase was especially significant in pTRV-*CCD7* and pTRV-*CCD8* plants (Fig. 2b). Moreover, an increased level of oxidized proteins was observed after 12 h of heat stress in the VIGS plants compared with that in the pTRV plants (Fig. 2c).

To substantiate the role of strigolactones in the heat response, we supplied the roots of wild-type plants with a GR24^5DS^ solution of 1, 3, or 9 µM or with distilled water containing an equal amount of acetone as the 1 µM GR24^5DS^ solution as a control at 24 h before the start of heat stress at 42 °C. After heat exposure for 48 h, the GR24^5DS^-treated WT plants exhibited reduced sensitivity to heat, as evidenced by the less severe wilting and lower REL values and MDA contents in the leaves than those in the leaves of control plants (Fig. S6a–c). Additionally, after 12 h of heat exposure, the accumulation of oxidized proteins triggered by heat stress was attenuated in the GR24^5DS^-treated plants, especially in the plants pretreated with GR24^5DS^ at 9 µM (Fig. S6d). Moreover, root application of GR24^5DS^ at different concentrations differentially induced the accumulation of HSP70 protein after 12 h of heat stress (Fig. S6e).

We then generated transgenic CRISPR-*ccd7* mutants, and homozygous *ccd7* T_2_ progenies with a 2 bp (TA) deletion were used. In comparison to WT plants, the *ccd7* mutant plants displayed increased lateral branches, decreased plant height, 58–63% less orobanchol, solanacol and didehydro-orobanchol, and higher stomatal conductance (Fig. S7). We then treated the roots of WT and *ccd7* plants with GR24^5DS^ at 3 µM at 24 h before they were exposed to heat stress at 42 °C. Heat-induced plant wilting was alleviated by the application of GR24^5DS^ in both WT and *ccd7* plants (Fig. S8a). Under optimal conditions, no significant differences were found in the actual quantum efficiency of PSII photochemistry (Ф_PSII_), the maximum quantum yield of PSII (*Fv/Fm*), and REL among these plants (Figs. 2d, e and S8b). Heat stress resulted in more significant decreases in Ф_PSII_ and *Fv/Fm*, as well as a more significant increase in REL in *ccd7* plants relative to the WT plants after 48 h of heat exposure. However, the values of these parameters did not significantly differ between the heat-exposed WT and *ccd7* plants in the presence of GR24^5DS^ (Figs. 2d, e and S8b). Moreover, less accumulation of the HSP70 protein was induced by heat stress in *ccd7* plants than in WT plants; however, application of GR24^5DS^ substantially increased the accumulation of HSP70 protein in both WT plants and *ccd7* plants after heat exposure for 12 h (Fig. 2f). Thus, it is likely that strigolactones induce heat stress tolerance in tomato, which is associated with an increased level of HSP70 protein in the leaves of plants.

### Strigolactones are crucial for the induction of the *CBF* transcript and the cold response

**Fig. 3:**
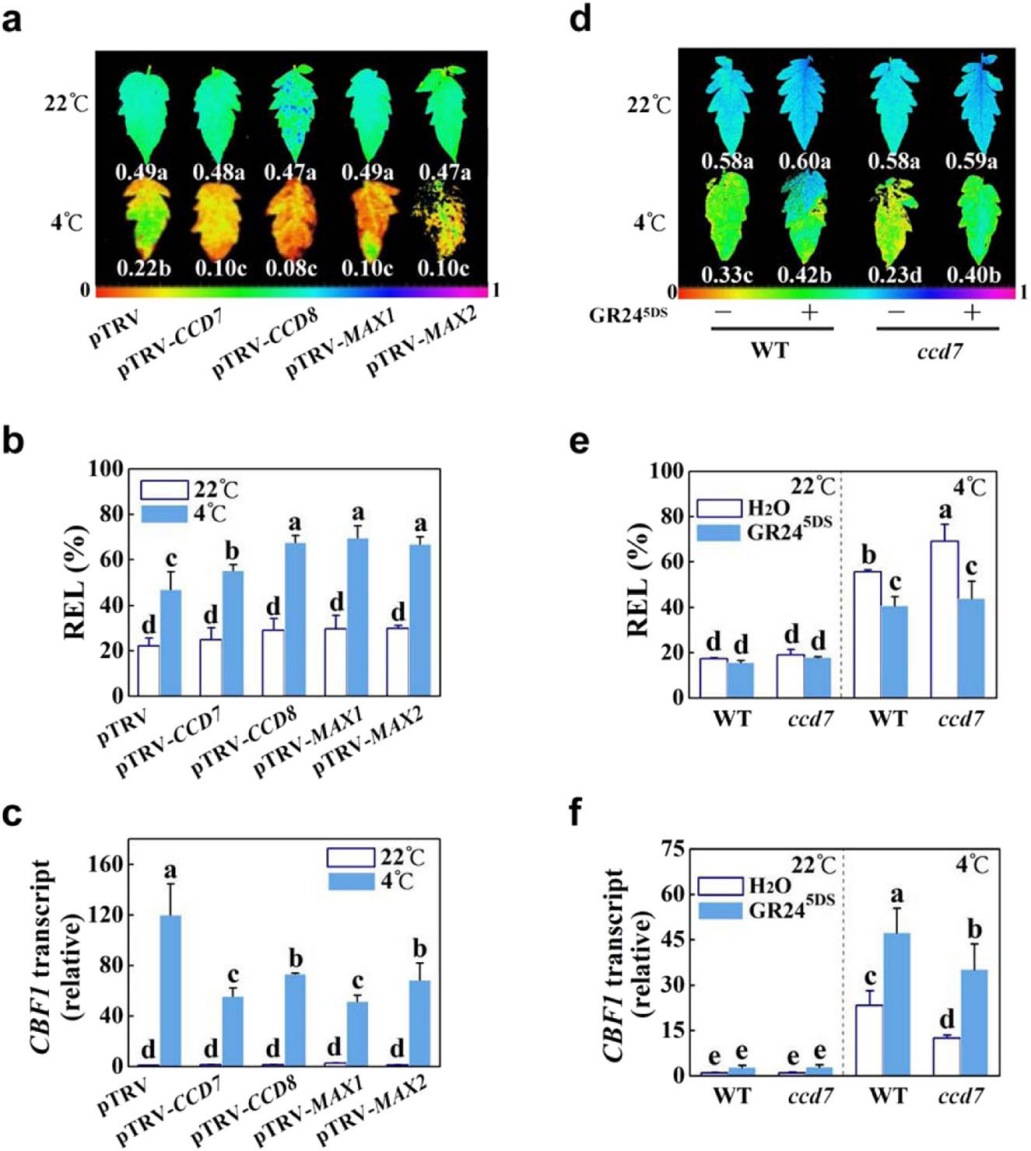
Effects of suppression of strigolactone biosynthesis and signaling and of GR24^5DS^ application on the cold response in tomato. **a**, **d** Actual quantum efficiency of PSII photochemistry (Ф_PSII_) of the leaves from plants after 7 d of cold at 4 °C. The color scale below the image ranges from 0 to 1.0 (purple). **b**, **e** Relative electrolyte leakage (REL) in the leaves of plants after 7 d of cold at 4 °C. **c**, **f** Transcript levels of *CBF1* in the leaves of plants after 12 h of cold at 4 °C. For (**a**–**c**), the plants used for gene silencing were the Ailsa Craig background. Plants transformed with the empty vector pTRV served as controls. For (**d**–**f**), WT refers to the wild type (Condine Red), and *ccd7* refers to transgenic CRISPR-*ccd7* mutants. A GR24^5DS^ (3 µM, 15 mL) solution was applied to the roots of each plant 24 h before cold stress at 4 °C. The plus and minus marks represent the application of GR24^5DS^ and water solution, respectively. The results in (**b**, **c**, **e**, **f**) are mean ± SD of three biological replicates; for (**a**) and (**d**), 15 leaves were used. Significant differences are indicated by different letters (*P* < 0.05, Tukey’s test)

To establish whether strigolactones also play a role in the cold response, 5-week-old *CCD7*-, *CCD8*-, *MAX1*-, and *MAX2*-silenced plants were exposed to cold conditions at 4 °C. After 7 days (d) of cold exposure, the pTRV plants exhibited slight wilting; in contrast, the VIGS plants showed severe wilting (Fig. S9a). At 22 °C, there were no differences in Ф_PSII_ (Fig. 3a) and *Fv/Fm* (Fig. S9b) among these plants. After 7 d of cold exposure, the leaves of the VIGS plants had lower *Fv/Fm* and Ф_PSII_ than the pTRV leaves (Figs. 3a and S9b). Moreover, exposure to cold resulted in more significant increases in the REL value and MDA accumulation in the VIGS plants than in the pTRV plants (Figs. 3b and S9c). Importantly, the cold-induced transcription of *CBF1*, which acts as a pivotal regulator in cold responses, was significantly attenuated in the leaves of VIGS plants (Fig. 3c).

We then applied GR24^5DS^ to the roots of plants and exposed these plants to cold conditions at 4 °C. In addition to severe wilting, leaves of *ccd7* plants showed lower *Fv/Fm* and Ф_PSII_ than those of the WT plants after cold stress in the absence of GR24^5DS^ (Figs. 3d and S10). However, there were no significant differences in Ф_PSII_ and *Fv/Fm* between the leaves of WT plants and *ccd7* plants when the roots were pretreated with GR24^5DS^ before the exposure of plants to cold stress (Figs. 3d and S10b). In agreement with the above results, the leaves of *ccd7* plants had higher REL and lower transcript levels of *CBF1* compared to those of the WT plants in the absence of GR24^5DS^ under cold conditions (Fig. 3e, f). Importantly, GR24^5DS^ application led to a significant decrease in REL and an increase in the transcript levels of *CBF1* in both *ccd7* mutants and WT plants after stress (Fig. 3e, f). Therefore, our results reveal that strigolactones act as positive regulators in cold tolerance and the induction of *CBF* transcript levels under cold stress.

### Strigolactones induce ABA biosynthesis and ABA-dependent transcriptional responses under heat and cold stresses

**Fig. 4:**
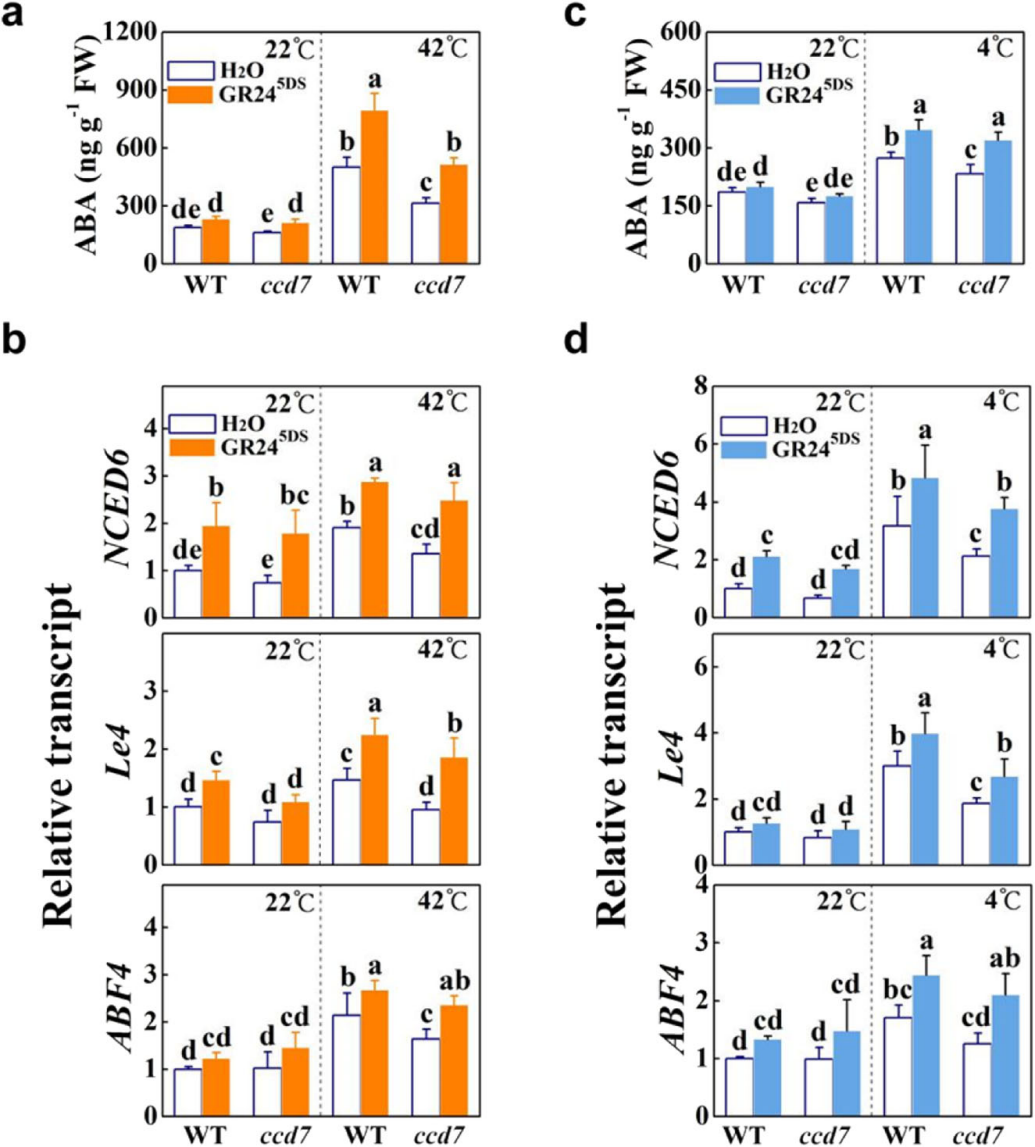
Effects of *CCD7* mutation and GR24^5DS^ application on heat- and cold-triggered ABA biosynthesis and ABA-dependent transcriptional responses in tomato. **a**, **c** ABA accumulation in the leaves of WT and *ccd7* plants with or without GR24^5DS^ treatment under heat and cold stresses. **b**, **d** Transcript levels of *NCED6*, *Le4*, and *ABF4* in the leaves of WT and *ccd7* plants with or without GR24^5DS^ application under hot and cold conditions. A GR24^5DS^ (3 µM, 15 mL) solution was applied to the roots of each plant 24 h before stress. WT refers to the wild type (Condine Red), and *ccd7* refers to transgenic CRISPR-*ccd7* mutants. Leaf tissues were collected 12 h after heating at 42 °C or cold stress at 4 °C. The results are mean ± SD of three biological replicates. Significant differences are indicated by different letters (*P* < 0.05, Tukey’s test)

ABA participates in the regulation of abiotic stresses, especially dehydration stress^37^. To explore whether ABA is associated with strigolactone-induced heat or cold responses in tomato, we determined the ABA content and the transcript accumulation of several genes related to ABA biosynthesis and signaling in the leaves of both *ccd7* plants and GR24^5DS^-treated plants. In the absence of stress, mutation of *CCD7* did not significantly alter ABA accumulation (Fig. 4a, c). Notably, the ABA content increased after heat or cold stress in both WT and *ccd7* plants, and this effect was more obvious with the application of GR24^5DS^ (Fig. 4a, c). Although heat and cold induced the accumulation of ABA in *ccd7* plants, their ABA content was still significantly lower than that in WT plants at 42 and 4 °C, respectively (Fig. 4a, c).

We next analyzed the transcript accumulation of genes involved in ABA biosynthesis and signaling. The mutation of *CCD7* did not significantly change the transcript levels of the ABA biosynthesis gene *9-CIS EPOXY CAROTENOID DIOXYGENASE 6* (*NCED6*) or of the ABA-responsive genes *Lycopersicon esculentum DEHYDRIN 4* (*Le4*) and *ABA-RESPONSIVE ELEMENT BINDING FACTOR 4* (*ABF4*) at 22 °C (Fig. 4b, d). Heat stress and cold stress both significantly upregulated the transcript levels of *NCED6*, *Le4*, and *ABF4* in WT and *ccd7* plants, except for the transcript levels of *Le4* in *ccd7* plants under hot conditions and *ABF4* in *ccd7* plants under cold conditions (Fig. 4b, d). Importantly, treatment with GR24^5DS^ increased the transcript levels of *NCED6*, *Le4*, and *ABF4* in both WT and *ccd7* plants under heat or cold stress conditions. However, transcript levels of these genes were always lower in *ccd7* mutants than in WT plants (Fig. 4b, d). This evidence demonstrates that strigolactones participate in modulating ABA biosynthesis and ABA-dependent transcriptional responses under heat and cold stress conditions.

### Strigolactones enhance antioxidant responses under heat and cold stress

**Fig. 5:**
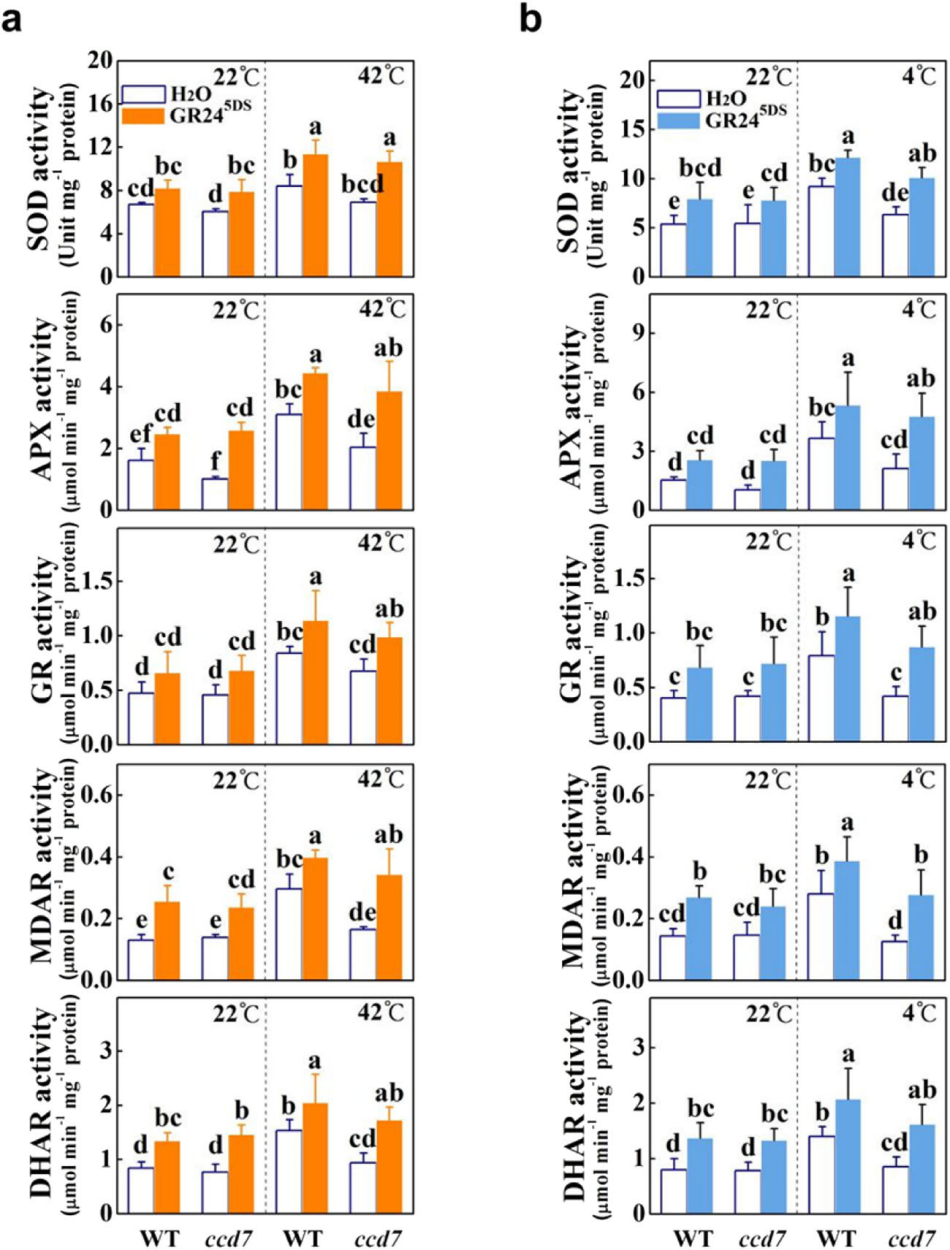
Effects of *CCD7* mutation and GR24^5DS^ application on heat- and cold-induced antioxidant responses in tomato. **a**, **b** Antioxidant enzyme activity in the leaves of WT and *ccd7* plants treated or not treated with GR24^5DS^ under hot and cold conditions. The GR24^5DS^ (3 µM, 15 mL) solution was applied to the roots of each plant 24 h before stress. WT refers to the wild type (Condine Red), and *ccd7* refers to transgenic CRISPR-*ccd7* mutants. Leaf tissues were collected after 12 h of heating at 42 °C (**a**) or cold stress at 4 °C (**b**). The results are mean ± SD of three biological replicates. Significant differences are indicated by different letters (*P* < 0.05, Tukey’s test)

To determine whether strigolactones are associated with antioxidant responses under heat and cold stresses, we determined hydrogen peroxide (H_2_O_2_) accumulation in the leaves after 48 h of heat stress or 7 d of cold stress. After heat or cold exposure, *ccd7* mutants accumulated more H_2_O_2_ than WT plants. However, root application of GR24^5DS^ significantly reduced H_2_O_2_ concentrations in both WT and *ccd7* plants under stress conditions (Fig. S11). Then, we measured the antioxidant enzyme (SOD, APX, GR, MDAR, and DHAR) activity in WT and *ccd7* plants after 12 h of heat or cold stress. At 22 °C, no obvious differences in the activity of these enzymes were found between WT and *ccd7* plants (Fig. 5). Their activity increased significantly under heat and cold stresses in WT plants but not in *ccd7* plants. Finally, root application of GR24^5DS^ significantly increased their activity under optimal growth conditions or stress conditions in both WT and *ccd7* plants (Fig. 5). Consistent with the changes in the activity of these enzymes, no obvious differences were found in the transcript levels of *Cu/Zn-SOD*, *APX*, *GR*, *MDAR*, and *DHAR* between WT and *ccd7* plants under optimal conditions (Fig. S12). While heat and cold stresses significantly induced the transcription of these genes in WT plants, such inductions were not observed in *ccd7* plants (Fig. S12). Similarly, root application of GR24^5DS^ significantly increased the transcript levels of *Cu/Zn-SOD*, *APX*, *GR*, *MDAR*, and *DHAR* under optimal or stress conditions in both WT and *ccd7* plants (Fig. S12). Thus, these data reveal that strigolactones are generally involved in the regulation of the enzymatic antioxidant response under both optimal and stress conditions in tomato plants.

### ABA mediates strigolactone-induced heat and cold stress responses

**Fig. 6:**
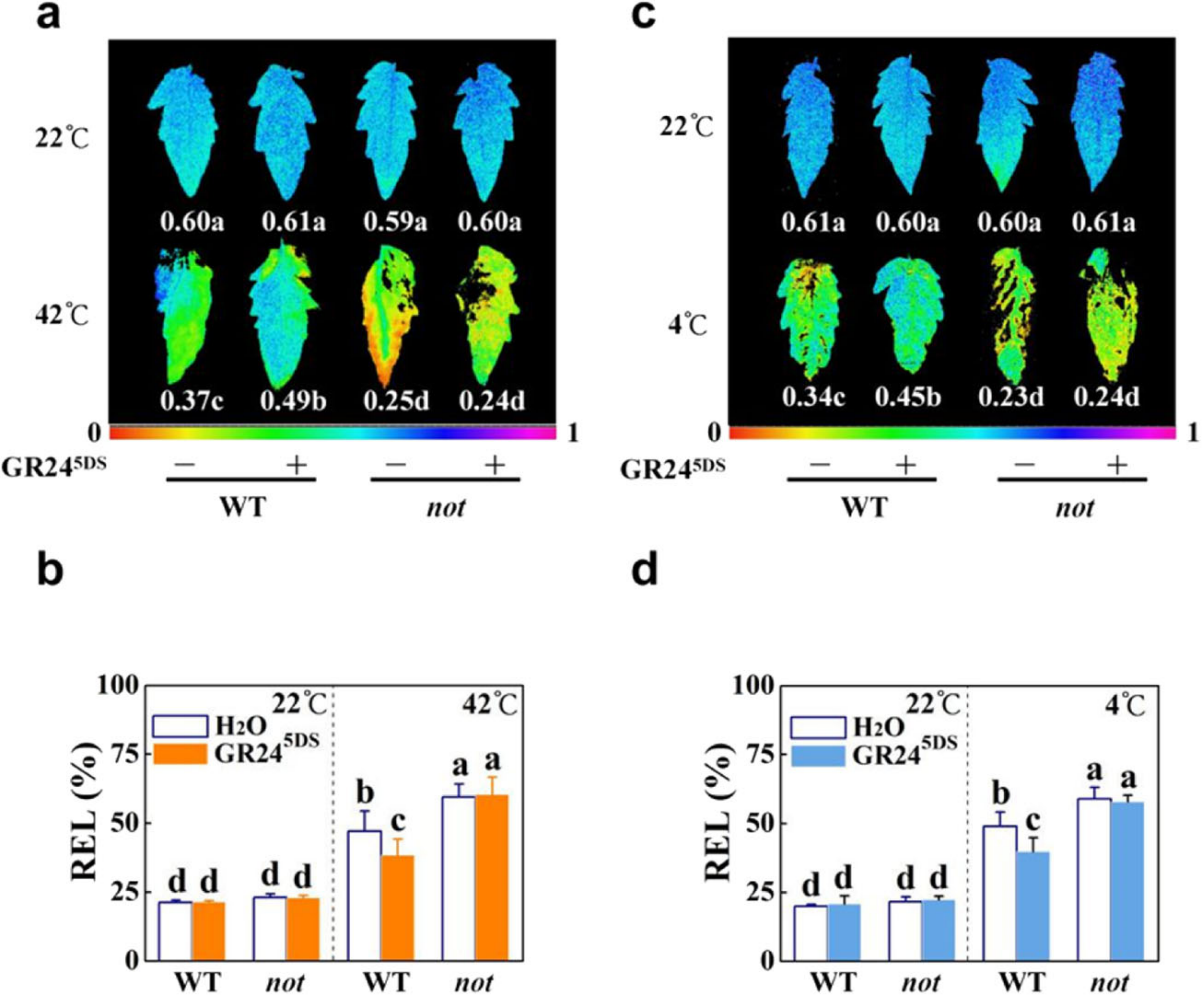
Effects of GR24^5DS^ on heat and cold tolerance in wild-type (WT; Ailsa Craig) and ABA-deficient mutant *not* plants. **a**, **c** Changes in the actual quantum efficiency of PSII photochemistry (Ф_PSII_) in leaves with or without GR24^5DS^ application after 48 h of heat stress at 42 °C (**a**) or 7 d of cold stress at 4 °C (**c**). The color scale below the image ranges from 0 to 1.0 (purple). **b**, **d** Relative electrolyte leakage (REL) in leaves with or without GR24^5DS^ application after 48 h of heat at 42 °C (**b**) or 7 d of cold at 4 °C (**d**). The GR24^5DS^ (3 µM, 15 mL) solution was applied to the roots of each plant 24 h before stress. The results in (**b**) and (**d**) are mean ± SD of three biological replicates; for (**a**) and (**c**), 15 leaves were used, and the plus and minus marks represent the application of GR24^5DS^ and water solution, respectively. Significant differences are indicated by different letters (*P* < 0.05, Tukey’s test)

**Fig. 7:**
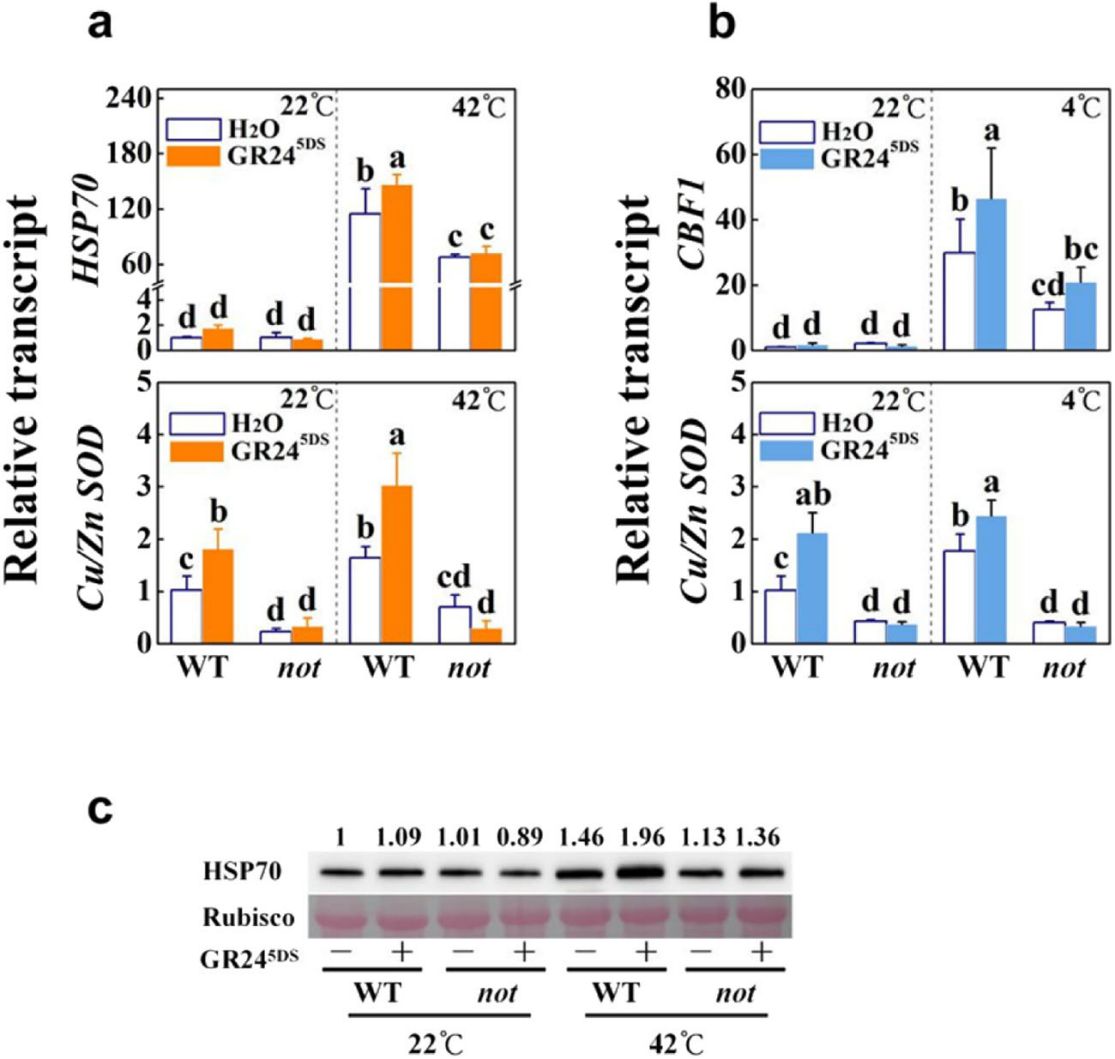
Effects of GR24^5DS^ on heat- and cold-responsive genes and HSP70 protein accumulation in wild-type (WT; Ailsa Craig) and ABA-deficient mutant *not* plants. **a** Transcript levels of *HSP70* and *Cu/Zn-SOD* in leaves of plants treated or untreated with GR24^5DS^ after 12 h of heat stress at 42 °C. **b** Transcript levels of *CBF1* and *Cu/Zn-SOD* genes in leaves of plants treated or untreated with GR24^5DS^ after 12 h of cold stress at 4 °C. **c** HSP70 protein accumulation in the leaves of plants treated or untreated with GR24^5DS^ after 12 h of heat stress at 42 °C. The number above each lane indicates the relative band intensity value. The GR24^5DS^ (3 µM, 15 mL) solution was applied to the roots of each plant 24 h before the heat or cold stress treatment. The plus and minus marks represent the application of GR24^5DS^ and water solution, respectively. For (**a**) and (**b**), the results are mean ± SD of three biological replicates. Significant differences are indicated by different letters (*P* < 0.05, Tukey’s test)

We next determined the effects of GR24^5DS^ on heat and cold responses in the ABA-deficient mutant *notabilis* (*not*). After 48 h of heat exposure or 7 d of cold exposure, most of the leaves on the *not* plants showed severe wilting symptoms (Fig. S13a, c) accompanied by decreased values of Ф_PSII_ and increased REL relative to those in the WT plants (Fig. 6a–d). While GR24^5DS^ significantly increased the values of Ф_PSII_ and decreased the values of REL in the WT plants, such effects were abolished in the *not* plants after heat (Fig. 6a, b) or cold treatment (Fig. 6c, d). After 12 h of heat exposure, heat-induced ABA accumulation and transcription of *HSP70*, *HSP90*, *Cu/Zn-SOD*, *APX*, *GR*, *MDAR*, *DHAR*, *Le4*, and *ABF4* were substantially attenuated in the *not* plants (Figs. 7a, S13b, and S14a, b). Similarly, ABA accumulation and transcription of *CBF1*, *CBF3*, *Cu/Zn-SOD*, *APX*, *GR*, *MDAR*, *DHAR*, *Le4*, and *ABF4* were significantly compromised in the *not* plants after 12 h of cold stress (Figs. 7b, S13d, and S14c, d). Moreover, GR24^5DS^ failed to induce the accumulation of ABA and the transcription of *HSP70*, *HSP90*, antioxidant genes, and ABA-responsive genes in response to heat and that of *CBF1*, *CBF3*, antioxidant genes, and ABA-responsive genes in response to cold stress, respectively, in *not* mutants (Figs. 7a, b, S13b, d, and S14). Similarly, the effect of heat and GR24^5DS^ on the induction of the HSP70 protein was less noticeable in *not* plants (Fig. 7c). Thus, ABA mediates at least a subset of strigolactone-induced heat and cold stress responses.

## Discussion

Recently, strigolactones have been found to positively modulate dark chilling resistance in pea and Arabidopsis^35^ and cold tolerance in rice^17^, and alleviate heat-induced adverse effects on leaf growth in tall fescue^36^. Several studies have demonstrated the critical role of strigolactones in the drought response in Arabidopsis, *L.* *japonicus*, and tomato^26,30–32^ and the induction of *HSP* transcript levels in Arabidopsis^26^. In this study, we demonstrate that strigolactones act as positive regulators in plant responses to heat and cold stresses in tomato. Importantly, we found that strigolactone-induced tolerance against heat and cold stresses is linked to the induction of CBFs, HSPs and antioxidant metabolism in a largely ABA-dependent manner.

### Strigolactones play a positive role in both heat and cold responses in tomato

Studies have revealed that plants deficient in strigolactone biosynthesis or signaling-related genes show decreased stress tolerance in several species, while application of a synthetic strigolactone analog increases tolerance to drought stress in Arabidopsis and wheat^17,26,27,31,35,38,39^. Here, multiple lines of evidence were provided to indicate that strigolactones positively regulate responses to heat and cold stresses in tomato. First, the transcript accumulation of both strigolactone biosynthesis genes (*CCD7*, *CCD8*, *MAX1*) and the signaling gene *MAX2*, as well as the accumulation of solanacol, all increased under heat or cold stress in tomato roots (Fig. 1); however, strigolactone biosynthesis is repressed by osmotic-related stresses, such as drought and salinity, in the roots of nonmycorrhizal dicot plants, including tomato, lettuce, and *L.* *japonicus*^30,31,40,41^, implying that different environmental stimuli may have different effects on the regulation of strigolactone biosynthesis. In addition, the transcriptional activation of the pathway in the leaves (Fig. S1) suggests that the observed phenotypes may be at least in part due to locally produced strigolactones; however, since the accumulation of strigolactones is very low and undetectable in the shoots of plants such as Arabidopsis and tomato^22,31^, this point cannot be solved analytically at this stage. Second, disruption of *CCD7* or silencing of *CCD7*, *CCD8*, *MAX1*, or *MAX2* enhanced the susceptibility to heat and cold stresses, as indicated by the increased values of REL and MDA in these plants relative to those in the control plants (Figs. 2, 3, S5, and S8–S10). Third, root application of the strigolactone analog GR24^5DS^ mitigated the damage against heat and cold stresses (Figs. 2, 3, and S6). These data strongly indicate that strigolactones are important in both heat and cold stress responses in tomato.

We found that mutation of *CCD7* decreased while application of GR24^5DS^ increased the accumulation of the HSP70 protein in response to heat stress (Fig. 2f). There is evidence that the transcript levels of *HSP* and *HEAT SHOCK TRANSCRIPTION FACTOR A6B* (*HSFA6B*) are responsive to strigolactones under stress^26^ or normal conditions^42^. HSPs can protect cellular proteins against severe injury under hot conditions, which is essential for plant survival under heat stress^9,11^. In agreement with this, an increased and a decreased accumulation of oxidized proteins were observed in the VIGS plants and the GR24^5DS^-treated WT plants, respectively, after exposure to heat stress (Fig. 2c and S6d). On the other hand, CBF1 plays a pivotal role in cold responses by inducing *COR* genes^7,12^. Chromatin immunoprecipitation sequencing (ChIP-seq) assays show that the transcription factor SUPPRESSOR OF MAX2-LIKE 6 (SMXL6) involved in strigolactone signaling directly binds to the *CBF* promoter^42^. In agreement with this, we found that silencing *CCD7*, *CCD8*, *MAX1* or *MAX2*, or disruption of *CCD7* partially compromised cold-induced transcript accumulation of *CBF1*, while application of GR24^5DS^ had the opposite effects on the WT and strigolactone-deficient plants (Fig. 3c, f). Therefore, strigolactones activate *CBF1* transcription in the cold response.

### ABA participates in strigolactone-induced heat and cold responses

ABA is known to be involved in plant responses to abiotic stresses such as salinity, drought, heat, and cold^5,15,37^. When encountering these stresses, plants accumulate ABA with the induction of stomatal closure and increase in antioxidant capacity^13,14^. ABA can crosstalk with other plant hormones, such as gibberellins and strigolactones, involved in plant growth, development, and stress responses^16,17^. Recently, several research groups have found that strigolactone-related mutants of Arabidopsis, *L.* *japonicus,* and tomato show decreased accumulation of ABA or exhibit increased water loss rates under dehydration and/or reduced ABA responsiveness compared with WT plants^26,27,30,31,38^. Interestingly, the rice *d3* (*dwarf3*), *d10*, and *d17* mutants are more drought tolerant, with increased ABA contents, compared to the WT plants, while the *d27* mutants exhibit reduced drought tolerance with decreased ABA accumulation^43^. The compromised stress tolerance observed in the *max2-3* mutant is linked to the downregulation of numerous stress- and/or ABA-responsive genes in Arabidopsis^26^. Moreover, the application of GR24 can induce the transcription of ABA catabolic genes and alleviate stress-induced seed dormancy^44–46^. Therefore, strigolactones can alter ABA homeostasis and ABA sensitivity. Consistent with previous studies in Arabidopsis and tomato^26,31^, silencing of strigolactone biosynthesis genes or *MAX2* resulted in increased water loss in detached leaves and higher stomatal conductance (Fig. S4). The increased stomatal conductance and sensitivity of pTRV-*MAX2* leaves to dehydration relative to pTRV-*CCD7*, pTRV-*CCD8*, and pTRV-*MAX1* leaves is probably due to the role of MAX2 linked not only to the strigolactone pathway but also to the (KARRIKIN INSENSITIVE 2) KAI2-dependent signaling^47^. Moreover, *ccd7* plants had decreased transcript levels of ABA biosynthesis gene and ABA-responsive genes *NCED6*, *Le4*, and *ABF4* with a decreased accumulation of ABA in the leaves under heat or cold stress conditions, while exogenous GR24^5DS^ treatment increased the transcription of these genes and induced ABA accumulation in both WT and *ccd7* plants (Fig. 4). In addition, we found that cold-induced transcription of *CBF1* and heat-induced HSP70 protein accumulation were linked to strigolactones and that these effects were partially ABA-dependent under heat or cold stress conditions (Figs. 2f, 3f, and 7); however, it remains to be studied how ABA biosynthesis is induced by strigolactones and how ABA regulates *CBF1* transcription and the HSP70 protein under temperature stresses.

### Strigolactones participate in the upregulation of the antioxidant system in response to heat and cold stresses

One of the findings of this study is the role of strigolactones in regulating the antioxidant system in stress responses. When suffering from stresses, plants accumulate ROS, which cause oxidative damage to cells and tissues^3^. In this study, tomato plants showed an increased accumulation of ROS, oxidized proteins, and MDA with decreases in *Fv*/*Fm* and Ф_PSII_ in the *ccd7* mutants or silenced plants after heat or cold exposure (Figs. 2, 3, and S8–S11). To protect cells against oxidative damage, plants activate ROS-scavenging systems, including antioxidant enzymes (DHAR, MDAR, SOD, GR, and APX)^3,4^. It seems likely that their activity is regulated by multiple hormones, such as ABA and brassinosteroids^14,48^. However, our understanding of the role of strigolactones in scavenging ROS is very limited. In rapeseed, GR24 application increases the activity of peroxidase and SOD and decreases lipid peroxidation under salt stress^49^. Moreover, treatment with GR24 enhances drought tolerance by increasing the activity of antioxidant enzymes (SOD, peroxidase, catalase, and APX) and decreasing lipid peroxidation^39^. Under heat and cold stresses, induction of both the activity of antioxidant enzymes (SOD, APX, GR, MDAR and DHAR) and the transcript levels of the corresponding genes was attenuated in *ccd7* mutants compared with those in WT plants, which is consistent with stronger oxidative stress in *ccd7* plants under heat and cold stresses (Figs. 5, S11–S12). Enzyme activity is regulated at several levels: gene transcription, protein turnover, and stability. In agreement with this, we found that the activity of antioxidant enzymes and/or the transcription of antioxidant genes were affected by the changes in the growth temperature and the strigolactone level (Figs. 5 and S12). These results suggest that strigolactones participate in the activation of ROS-scavenging systems. This conclusion is also substantiated by the results of the GR24^5DS^ application experiment (Figs. 5 and S12). Until now, both downregulation^50^ and upregulation^26,28^ of the transcript levels of many photosynthesis-related genes have been observed in strigolactone-related mutants. In our study, the alleviation of oxidative stress by strigolactones was followed by increased *Fv*/*Fm* and Ф_PSII_, suggesting that strigolactones may prevent stress-induced photoinhibition (*Fv*/*Fm*) and decrease of photosynthetic electron transport at PSII (Ф_PSII_) by alleviating oxidative stress. However, we could not exclude the direct role of strigolactones in the regulation of photosynthesis, as strigolactones have been found to potentially regulate light harvesting in tomato plants^50^.

As an antistress hormone, ABA enhances antioxidant capacity in response to stress^14^. Consistent with this, heat- and cold-induced transcription of the *Cu/Zn-SOD*, *APX*, *GR*, *MDAR*, and *DHAR* genes was compromised in the ABA-deficient mutant *not* (Figs. 7a, b and S13b, d). While GR24^5DS^ induced a significant increase in the transcript levels of *Cu/Zn-SOD*, *APX*, *GR*, *MDAR*, and *DHAR* in WT plants, this effect was again abolished in *not* plants (Figs. 7a, b and S13b, d). Therefore, the role of strigolactones in the activation of the antioxidant response in our study was partially dependent on ABA. Strigolactones are known to affect the sensitivity of guard cells to ABA in tomato, Arabidopsis, and *L.* *japonicus*^26,30,31^. Here, we found that the transcript levels of the ABA-responsive genes *Le4* and *ABF4* in ABA-deficient *not* plants did not increase in response to GR24^5DS^ under stress (Fig. S14b, d). Further study on the relationship of strigolactones and ABA is highly warranted.

## Materials and methods

### Plant materials

The tomato (*Solanum lycopersicum*) lines used in this study were the cultivar Ailsa Craig (wild type, WT), the ABA-deficient mutant *notabilis* (*not*; in the Ailsa Craig background)^51^, and Condine Red (WT). The tomato *ccd7* mutant on the Condine Red background was generated by a clustered, regularly interspaced, short palindromic repeat (CRISPR)/CRISPR-associated 9 (Cas9) technique^52^. The target sequence (ATTAACATTGCCTAGCCACG) was selected using the CRISPR-P program^53^ and subsequently synthesized, annealed and introduced into an AtU6-sgRNA-AtUBQ-Cas9 vector at the *Bbs*I site. Then, we inserted the reconstructed vectors into the pCAMBIA1301 binary vector at the *Hind*III and *Kpn*I sites. After confirmation by sequencing, the resulting *CCD7* CRISPR/Cas9 vector was transformed into *Agrobacterium tumefaciens* strain EHA105 and subsequently introduced into tomato seeds (Condine Red) according to a previously described method^54^. The homozygous line of *ccd7* mutants with a 2 bp deletion was identified and used (Fig. S7).

Virus-induced gene silencing (VIGS) experiments were carried out to silence the genes *CCD7*, *CCD8*, *MAX1*, and *MAX2* (Solyc12g010900.1.1, with a protein sequence similarity of 57% to the MAX2 protein in Arabidopsis)^55^. To specifically silence these target genes, 200~500 bp of the 3ʹ UTR was PCR-amplified with the specific primers listed in Table S1 and ligated into pTRV2 at the *Eco*RI and *Bam*HI sites. After sequencing confirmation, the reconstructed vector was electroporated into *A.* *tumefaciens* strain GV3101. The virus infection mediated by *Agrobacterium* was conducted as previously described^56^. Empty pTRV2 was also inoculated into tomato plants, which served as a control (pTRV). The plants were placed at 22/19 °C. Approximately 2~3 weeks later, root samples from the target-gene-silenced plants were collected to verify the silencing efficiency of their respective target genes with reverse transcription-quantitative PCR (RT-qPCR).

### Growth conditions and treatments

Seeds were germinated at 28 °C for 48 h, and then germinated seeds were grown in a mixture of vermiculite and peat (1:2, v/v). Growth conditions: 12 h photoperiod, 600 μmol m^−2^ s^−1^ photosynthetic photon flux density (PPFD), and 22/19 °C (day/night) temperature.

For the dehydration treatment, leaves of the *CCD7*-, *CCD8*-, *MAX1*-, and *MAX2*-silenced plants were detached and placed on a piece of weighing paper on a laboratory bench and weighed at the indicated time. Finally, the leaf water loss percentage (%) was determined. Stomatal conductance was determined on the fourth expanded leaves using an LI-6400 Portable Photosynthesis System (LI-COR, Lincoln, NE, USA). The PPFD was set at 1000 μmol m^−2^ s^−1^. Stomatal conductance was measured between 10:00 a.m. and 12:00 noon on six plants per silenced line or per treatment as reported by Liu et al.^30^. Both experiments were repeated three times, and each replication had six leaves.

For the strigolactone treatment, at 24 h before heat or cold stress, the roots of 5-week-old tomato plants were treated with GR24^5DS^ solution (StrigoLab S.r.l., Torino, Italy). A stock solution of GR24^5DS^ at 25 mM was dissolved in pure acetone (≥99.5%, AR, Sinopharm Chemical Reagent Co., Ltd.). After that, GR24^5DS^ was diluted to 1, 3, or 9 µM in distilled water for the heat response, in which water solution (distilled water containing an equal amount of acetone as the 1 µM GR24^5DS^ solution) served as a control (Fig. S6). For the other GR24^5DS^ treatments, a 3 µM concentration was adopted, and distilled water containing an equal amount of acetone was used as a control. A solution of 15 mL was applied to the roots of each plant.

### Analysis of heat and cold stress tolerance

For the heat or cold stress, 60~70 uniform 5-week-old plants for each genotype were divided into 6 groups and placed into 6 growth chambers (10~12 per chamber for each genotype) at 22/19 °C for 3 d. After that, the growth temperature was switched to a stable state of 42 °C (heat treatment) for 2 d or 4 °C (cold treatment) for 7 d in 3 growth chambers while another 3 growth chambers were maintained at 22 °C. The plants of each genotype were randomly placed with plants of other genotypes in the growth chambers.

REL in the leaves, which is an important index of cell membrane permeability, was determined as previously described^57^. The level of leaf lipid peroxidation was examined by determining the accumulation of MDA as described^58^. The actual quantum efficiency of PSII photochemistry (Ф_PSII_) and the maximum quantum yield of PSII (*Fv/Fm*) were detected via an IMAGING-PAM chlorophyll fluorometer (IMAG-MAXI; Heinz Walz, Germany) after a 30 min dark acclimation for the whole plants. The fifth leaf from the bottom was collected for assays.

### Purification of root extracts and germination bioassays

Root extracts were purified as described previously^29,59^. The seeds of *P.* *aegyptiaca* were obtained from Dr Jinxia Cui (Shihezi University, China). The bioassays of *P.* *aegyptiaca* seed germination were conducted according to our earlier study^59^.

### Detection of oxidized proteins and HSP70 protein by western blotting

The total soluble proteins of tomato leaves were determined according to our previous study^60^ via a BCA Protein Assay Kit (Pierce, USA). Oxidized protein levels in the soluble protein fraction were measured via an OxyBlot protein oxidation detection kit (Chemicon International, USA). The HSP70 protein samples were separated by 10% SDS-PAGE and examined using an anti-HSP70 polyclonal antibody (Beijing Protein Innovation Co., Ltd., China) and a horseradish peroxidase-linked secondary antibody (Cell Signaling Technology, USA). Then, the antigen–antibody signal was observed via a chemiluminescence kit (Perkin Elmer, USA) in accordance with the manufacturer’s protocol.

### Measurements of hydrogen peroxide (H_2_O_2_) levels

To detect the accumulation of leaf H_2_O_2_, 0.3 g fresh tissue was homogenized in 3 mL 1 M HClO_4_ according to a previously described method^61^.

### Assays of antioxidant enzyme activity

To detect the enzyme activity, 0.3 g leaf tissue was homogenized with 2 mL 50 mM phosphate buffer (pH 7.8) containing 2 mM L-ascorbic acid, 2% (w/v) polyvinylpyrrolidone K30, and 0.2 mM EDTA. After centrifugation at 12,000*g* for 20 min, supernatants were collected for analysis. The activity of SOD was measured by detecting the capacity to suppress the photochemical reduction of nitroblue tetrazolium (NBT) according to Stewart and Bewley^62^. The enzyme activity that results in a 50% inhibition of NBT photochemical reduction is defined as one unit. The activities of APX and DHAR were detected by a decrease and an increase at A_290_ and A_265_, respectively, and separately, following a previously described method^63^. The activities of GR and MDAR were both assayed by a decrease at A_340_ following previous protocols^64,65^. The units of enzyme (APX, DHAR, GR, and MDAR) activities were calculated according to Noctor et al.^66^. Spectrophotometric experiments were carried out on a UV-2410PC spectrophotometer (Shimadzu, Japan).

### Phytohormone measurements

For strigolactone measurements, 0.5 g frozen roots were homogenized with 500 µL 40% acetone/water. The homogenate was centrifuged at 12,000*g* for 5 min at 4 °C, and then the supernatant was removed. Afterward, the remaining pellet was eluted with 500 µL 50% acetone/water and centrifuged again. The analysis and quantification of strigolactones were performed as described previously^29,59^.

For ABA measurements, leaves were sampled for quantification at 12 h after exposure to heat or cold stress. Phytohormone extraction from tomato leaves and ABA analysis were conducted following the methods of Wang et al.^7^. Generally, 0.1 g frozen tomato leaves were extracted with 1 mL ethyl acetate, in which the internal standard D_6_-ABA (CDN Isotopes Inc., Canada) was added. After centrifugation at 18,000*g* for 10 min at 4 °C, the resulting pellet was once again extracted with 1 mL ethyl acetate. Supernatants collected twice were merged and evaporated to dryness under N_2_ gas. The residue was then resuspended in 500 µL 70% methanol (v/v) and centrifuged. Finally, the supernatant was analyzed via a liquid chromatography tandem mass spectrometry system (Varian 320-MS LC/MS, Agilent Technologies, the Netherlands).

### RNA isolation and quantitative PCR (qPCR) analysis

Total RNA was isolated from the tomato leaves or roots with an RNA extraction kit (Tiangen, Beijing, China) following the manufacturer’s protocol. Total RNA (1 µg) was reverse transcribed to the cDNA template via a ReverTra Ace qPCR RT Kit (Toyobo, Osaka, Japan). qPCR was conducted using a LightCycler 480 detection machine (Roche, Basel, Switzerland). qPCR procedures: denaturation at 95 °C for 3 min, followed by 45~50 cycles of denaturation at 95 °C for 30 s, annealing at 57 °C for 20 s, and extension at 72 °C for 30 s. The *ACTIN* gene, which is suitable for heat^67^ and cold^7^ responses in tomato, was used as an internal reference. Specific primers are shown in Table S2. The relative expression of genes was analyzed according to the methods of Livak and Schmittgen^68^.

### Statistical analysis

There were three replicates, and each replicate within a growth chamber consisted of 10~12 plants. For the determination of *Fv/Fm* and Ф_PSII_, 15 leaves from 10 independent plants were used (*n* = 15). For other measurements, at least three biological samples were used. Statistical analysis of bioassays was conducted using Statistics Analysis System (SAS) software, version 8 (SAS Institute). The means were analyzed by using Tukey’s test (*P* < 0.05), except for pairwise comparisons, in which Student’s *t*-test was adopted (*P* < 0.05).

## Accession numbers

Sequence data in this study are available in the Sol Genomics Network (https://solgenomics.net/) according to the following accession numbers: *ACTIN* (Solyc11g005330), *CCD7* (Solyc01g090660), *CCD8* (Solyc08g066650), *MAX1* (Solyc08g062950), *MAX2* (Solyc12g010900), *Cu/Zn-SOD* (Solyc11g066390), *APX* (Solyc01g111510), *GR* (Solyc09g091840), *MDAR* (Solyc08g081530), *DHAR* (Solyc05g054760), *CBF1* (Solyc03g026280), *CBF3* (Solyc03g026270), *HSP70* (Solyc04g011440), *HSP90* (Solyc12g015880), *NCED6* (Solyc05g053530), *Le4* (Solyc02g084850), and *ABF4* (Solyc11g044560).

## Supplementary information

Strigolactone manuscript-supplemental material.
