# Supplementary material for: Strigolactones positively regulate abscisic acid-dependent heat and cold tolerance in tomato
Source: Hortic Res. 2021 Nov 1;8:237. doi: 10.1038/s41438-021-00668-y (PMC8558334; doi:10.1038/s41438-021-00668-y)
Supplement: Supplementary file 1 — Strigolactone manuscript-supplemental material. [file 41438_2021_668_MOESM1_ESM.pdf]

Supporting Information

Article title: Strigolactones positively regulate abscisic acid-dependent heat and cold tolerance in tomato

Authors: Cheng Chi, Xuechen Xu, Mengqi Wang, Hui Zhang, Pingping Fang, Jie Zhou, Xiaojian Xia, Kai Shi, Yanhong Zhou & Jingquan Yu

The following Supporting Information is available for this article:

Fig. S1 Transcript levels of strigolactone biosynthetic genes in tomato leaves under heat and cold stresses.

Fig. S2 Silencing of strigolactone biosynthesis and signaling genes differentially altered *Phelipanche aegyptiaca* seed germination and strigolactone accumulation in tomato roots.

Fig. S3 Phenotypes of *CCD7*-, *CCD8*-, *MAX1*- and *MAX2*-silenced plants.

Fig. S4 Dehydration in leaves of *CCD7*-, *CCD8*-, *MAX1*- and *MAX2*-silenced plants.

Fig. S5 Phenotypes of suppression of strigolactone biosynthesis and signaling in response to heat stress.

Fig. S6 Effects of $GR24^{5DS}$ on heat responses and HSP70 protein accumulation.

Fig. S7 Identification of tomato *ccd7* CRISPR transgenic plants.

Fig. S8 Phenotypes of *ccd7* plants with or without $GR24^{5DS}$ application after heat stress.

Fig. S9 Effects of suppression of strigolactone biosynthesis and signaling on cold tolerance.

Fig. S10 Phenotypes of *ccd7* plants with or without $GR24^{5DS}$ application after cold stress.

Fig. S11 Effects of *CCD7* mutation and $GR24^{5DS}$ application on the accumulation of hydrogen peroxide in response to heat and cold stresses.

Fig. S12 Effects of *CCD7* mutation and $GR24^{5DS}$ application on heat- and cold-induced antioxidant genes in tomato.

Fig. S13 Effects of $GR24^{5DS}$ on heat- and cold-responsive and antioxidant genes in the ABA-deficient mutant *not*.

Fig. S14 Effects of $GR24^{5DS}$ on heat- and cold-triggered ABA biosynthesis and ABA-dependent transcriptional responses in the ABA-deficient mutant *not*.

Table S1 PCR primers for VIGS vector construction.

Table S2 Primers used for qPCR assays.

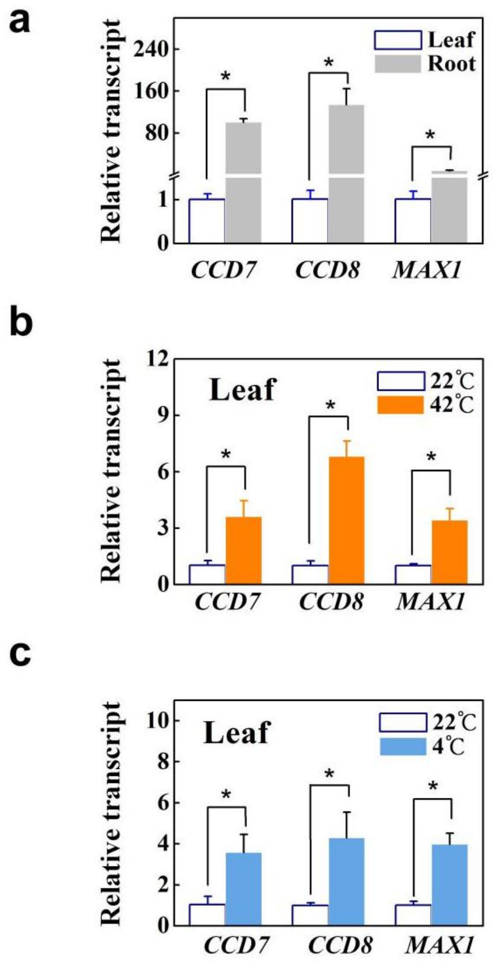

Fig. S1 Transcript levels of strigolactone-biosynthetic genes in tomato leaves under heat and cold stresses. Transcript levels of *CCD7*, *CCD8* and *MAX1* in tomato roots under normal conditions (**a**), and transcript levels of these genes in leaves in response to heat (**b**) or cold (**c**) stress. Gene transcript level was expressed as fold-change value over each specific gene (*CCD7*, *CCD8* and *MAX1*) in the leaves under normal conditions, which was defined as 1. Leaf tissues were collected after 6 h of heat stress at 42°C or cold stress at 4°C. Plants used for the experiments were the wild-type (WT; Condine Red) plants. The results are the means ± SD of three biological replicates. Significant differences are indicated by asterisks ($P < 0.05$, Student's t-test).

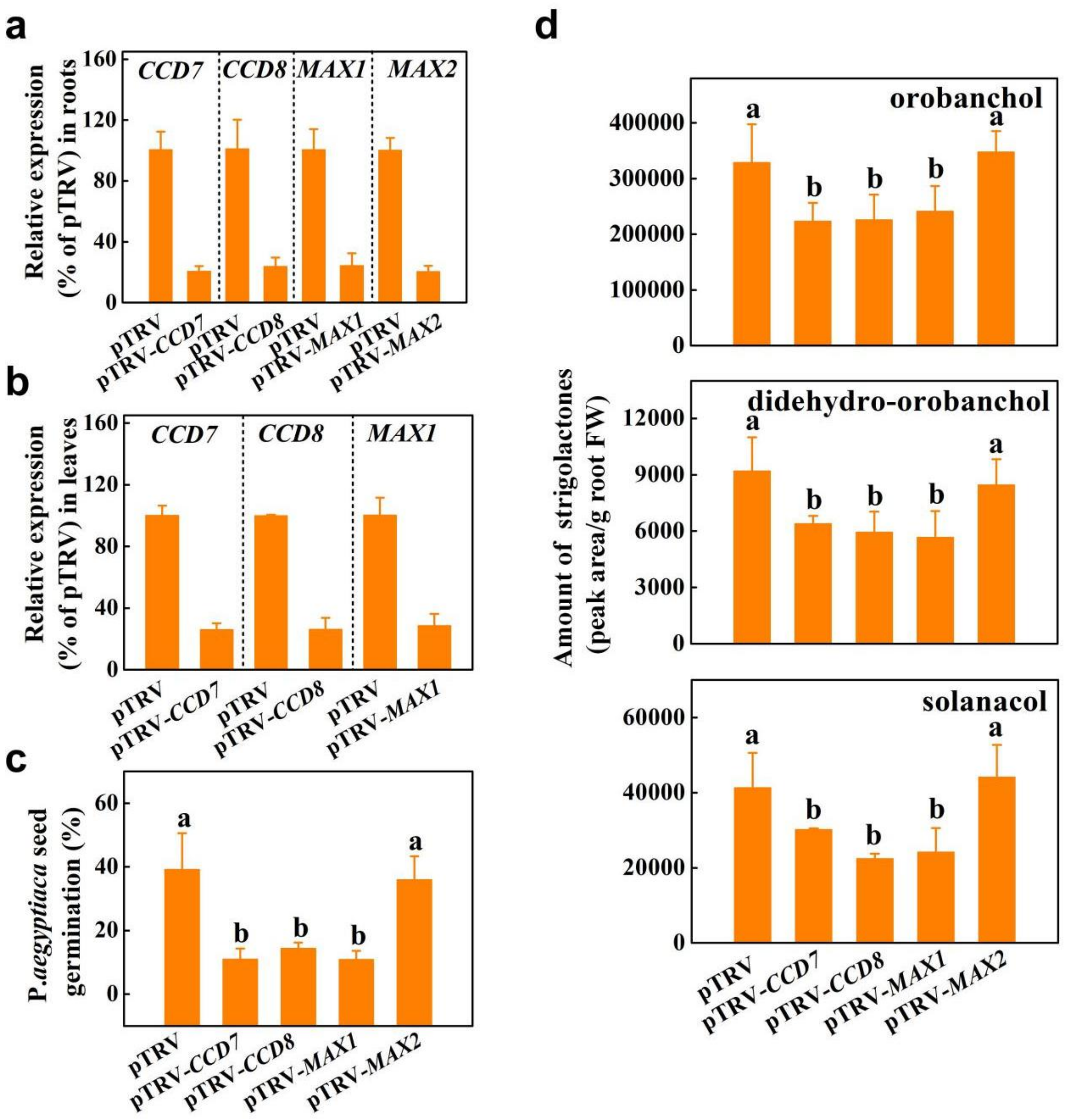

Fig. S2 Silencing of strigolactone biosynthesis and signaling genes differentially altered *Phelipanche aegyptiaca* seed germination and strigolactone accumulation in tomato roots. a Relative expression of *CCD7*, *CCD8*, *MAX1* and *MAX2* in VIGS plant roots. **b** Relative expression of *CCD7*, *CCD8* and *MAX1* in VIGS plant leaves. The levels were presented as percentages compared with that of the control pTRV plants, which were defined as 100%. **c** *Phelipanche aegyptiaca* seed germination by the induction of the root extracts from *CCD7*-, *CCD8*-, *MAX1*- and *MAX2*-silenced plants compared with the empty vector pTRV plants. **d** Accumulation of strigolactones in the root extracts of VIGS plants. The plants used for gene silencing were the Ailsa Craig background. The results are the means ± SD of three biological replicates. Significant differences are indicated by different letters ($P < 0.05$, Tukey's test).

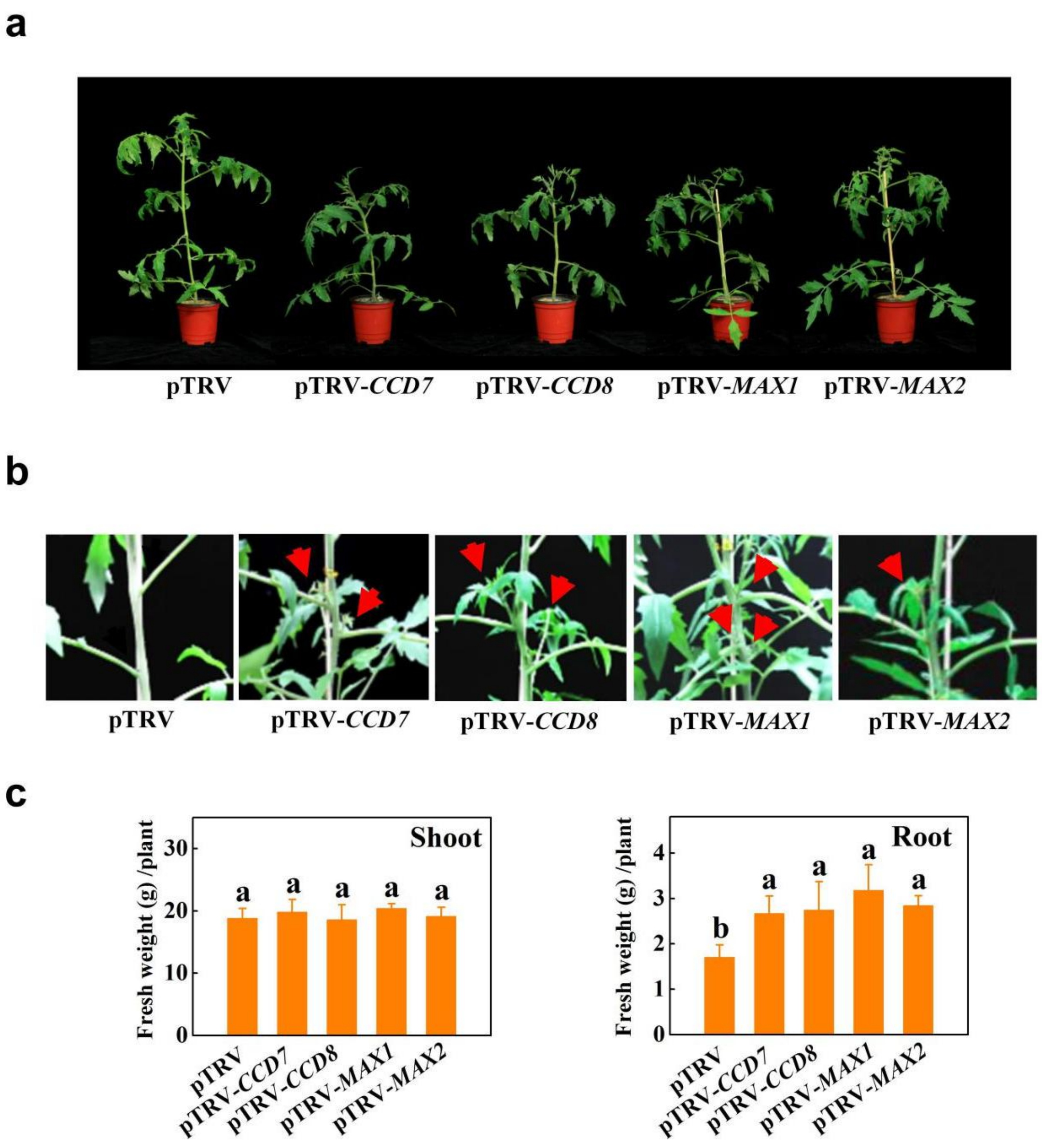

Fig. S3 Phenotypes of *CCD7*-, *CCD8*-, *MAX1*- and *MAX2*-silenced plants. a Phenotypes of plants at 6~7-leaf stage. **b** Phenotypes of plants at 10~12-leaf stage. **c** Shoot and root fresh weight for plants at 10-leaf stage. The plants used for gene silencing were the Ailsa Craig background. The results are the means ± SD of five biological replicates. Significant differences are indicated by different letters ($P < 0.05$, Tukey's test).

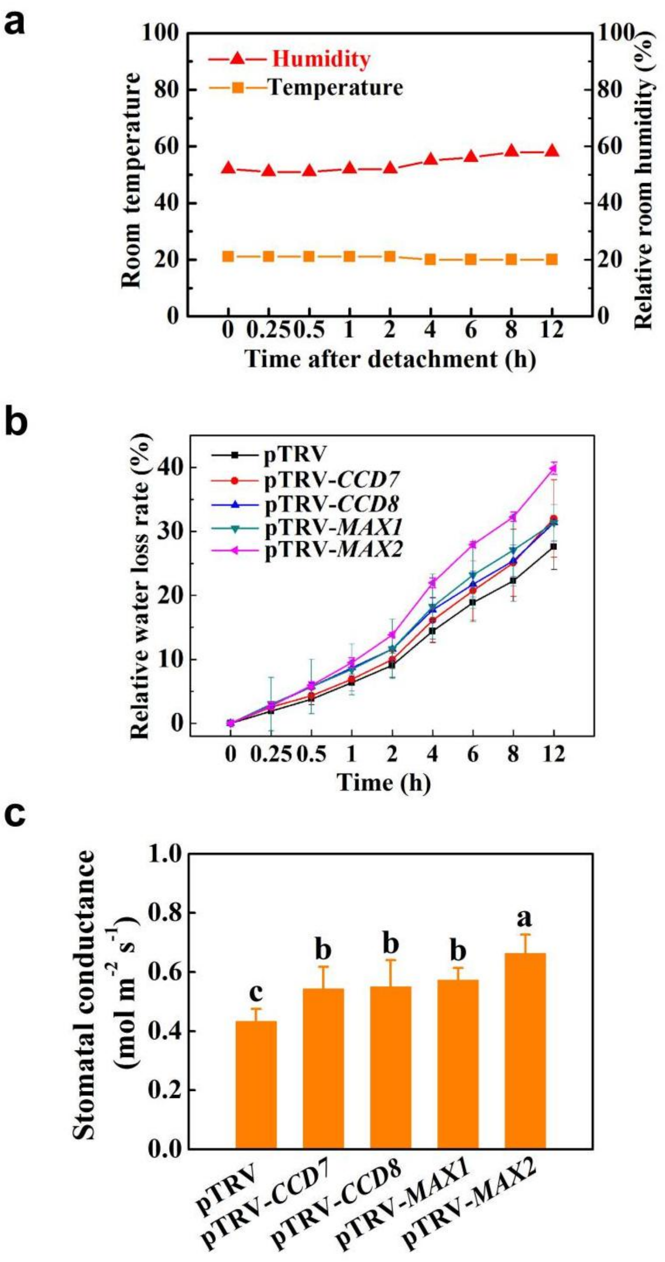

Fig. S4 Dehydration in leaves of *CCD7*-, *CCD8*-, *MAX1*- and *MAX2*-silenced plants. a Ambient temperature and humidity during the experiment. **b** Time course of relative leaf water loss rate (%) of strigolactone-depleted and -insensitive plants and the control pTRV plants exposed to dehydration stress. **c** Stomatal conductance of strigolactone-depleted and -insensitive plants and the control pTRV plants. The plants used for gene silencing were the Ailsa Craig background. The results are the means ± SD of six biological replicates. Significant differences are indicated by different letters ($P < 0.05$, Tukey's test).

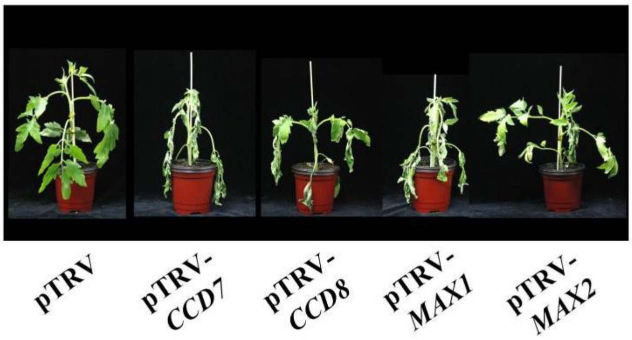

Fig. S5 Phenotypes of suppression of strigolactone biosynthesis and signaling in response to heat stress. The *CCD7*-, *CCD8*-, *MAX1*- and *MAX2*-silenced plants were subjected to heat stress at 42°C for 48 h. Plants used for gene silencing were the Ailsa Craig background.

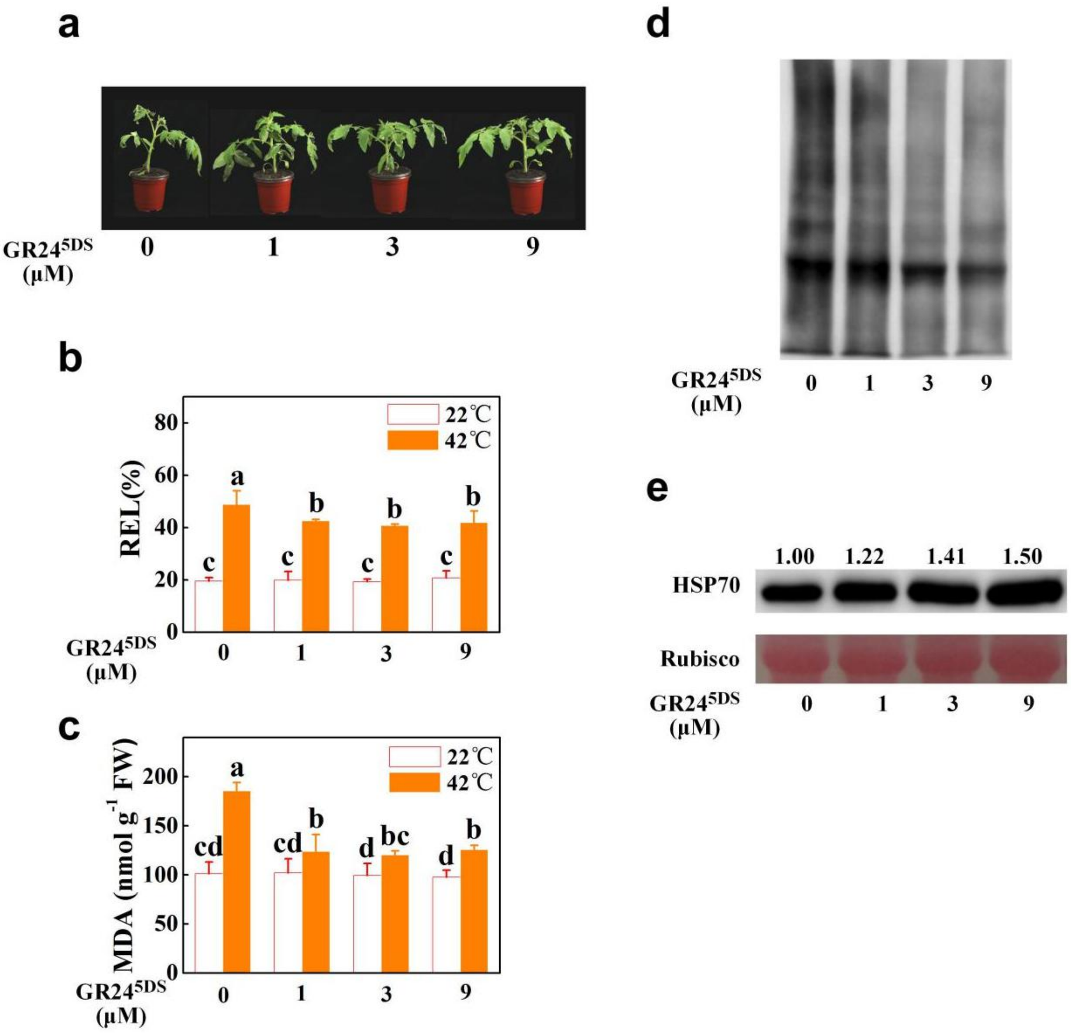

Fig. S6 Effects of GR24^{5DS} on heat responses and HSP70 protein accumulation. a Phenotypes of plants after 48 h of heat stress at 42°C. **b** Relative electrolyte leakage (REL) in the leaves after 48 h of heat stress at 42°C. **c** Malondialdehyde (MDA) accumulation in the leaves after 48 h of heat stress at 42°C. **d** Oxidized proteins in the leaves after 12 h of heat stress at 42°C. **e** HSP70 protein accumulation in the leaves after 12 h of heat stress at 42°C. The GR24^{5DS} solution (1 μM, 3 μM or 9 μM, 15 mL) was applied to the roots of each plant 24 h before the heat stress. The number above each lane indicates the relative band intensity value. The wild type (WT; Ailsa Craig) plants were used for GR24^{5DS} treatment. Water solutions (distilled water containing an equal amount of acetone as the 1 μM GR24^{5DS} solution) were used as controls. The results in **b** and **c** are the means ± SD of three biological replicates. Significant differences are indicated by different letters ($P < 0.05$, Tukey's test).

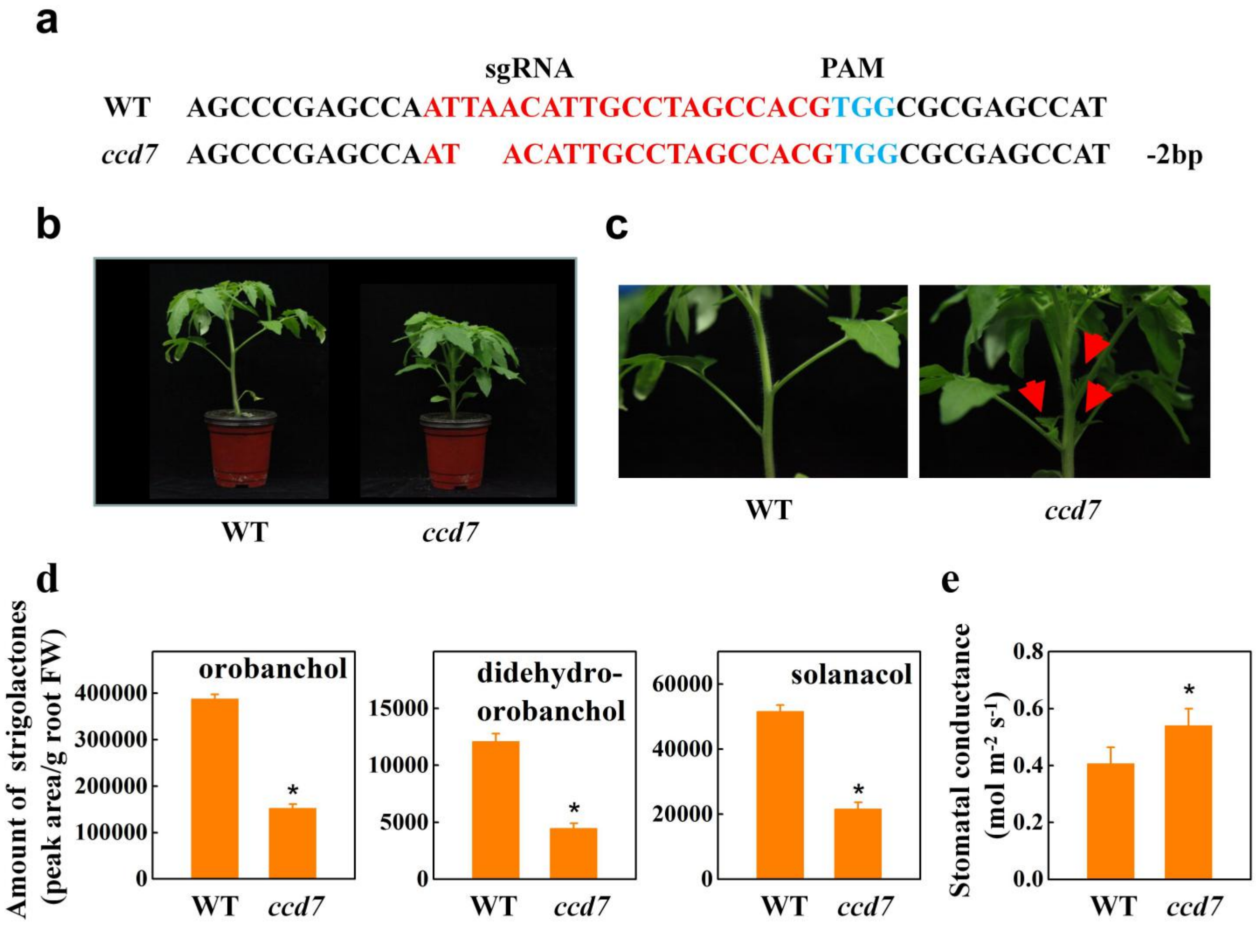

Fig. S7 Identification of tomato *ccd7* CRISPR transgenic plants. a sgRNA and mutation site of the tomato *ccd7* CRISPR transgenic plants. **b**, **c** Phenotypes of the tomato *ccd7* CRISPR transgenic plants at 5~6-weeks old. **d** Accumulation of strigolactones in the root extracts of *ccd7* plants. **e** Stomatal conductance in the leaves of *ccd7* plants. For **d** and **e**, the results are the means ± SD of three (d) or six (e) biological replicates. Significant differences are indicated by asterisks ($P < 0.05$, Student's t-test).

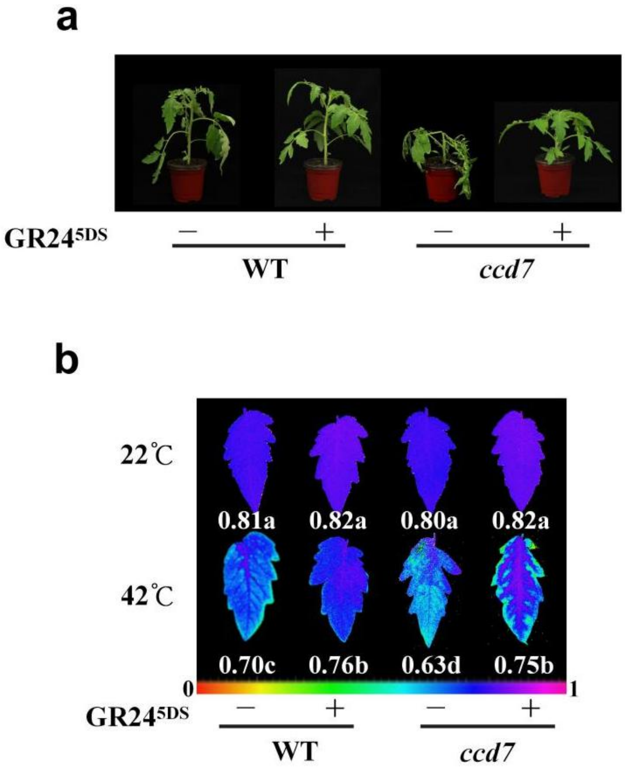

Fig. S8 Phenotypes of *ccd7* plants with or without GR24^{5DS} application after heat stress. a Phenotypes of plants after 48 h of heat stress at 42°C. **b** The maximum quantum yield of PSII (*Fv/Fm*) of the leaves from plants after 48 h of heat stress at 42°C. The GR24^{5DS} solution (3 μM, 15 mL) was applied to the roots of each plant 24 h before the heat stress. WT refers to the wild type (Condine Red), and *ccd7* refers to transgenic CRISPR-*ccd7* mutants. The plus and minus marks represent the application of GR24^{5DS} and water solution, respectively. For **b**, 15 leaves were used. Significant differences are indicated by different letters ($P < 0.05$, Tukey's test).

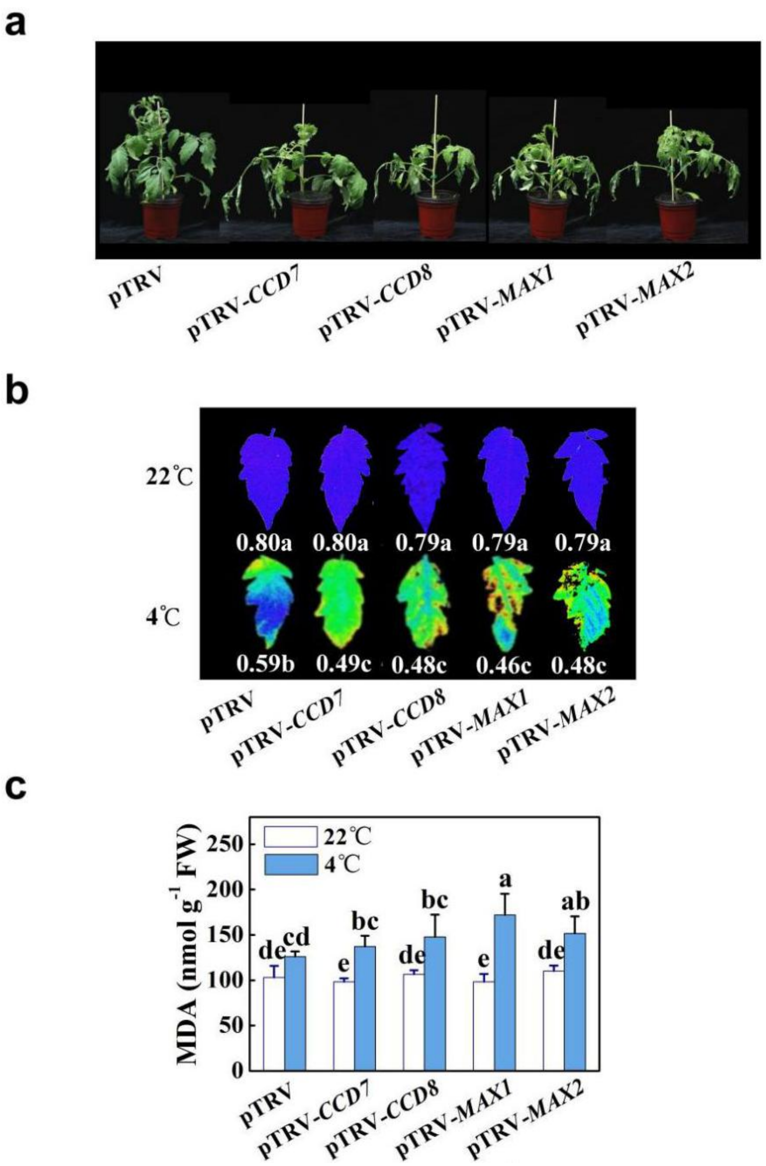

Fig. S9 Effects of suppression of strigolactone biosynthesis and signaling on cold tolerance. a Phenotypes of *CCD7*-, *CCD8*-, *MAX1*- and *MAX2*-silenced plants after 7 d of cold stress at 4°C. **b** The maximum quantum yield of PSII (*Fv/Fm*) of the leaves from plants after 7 d of cold stress at 4°C. **c** Malondialdehyde (MDA) accumulation in the leaves after 7 d of cold stress at 4°C. Plants used for gene silencing were the Ailsa Craig background. For **b**, 15 leaves were used. For **c**, the results are the means ± SD of three biological replicates. Significant differences are indicated by different letters ($P < 0.05$, Tukey's test).

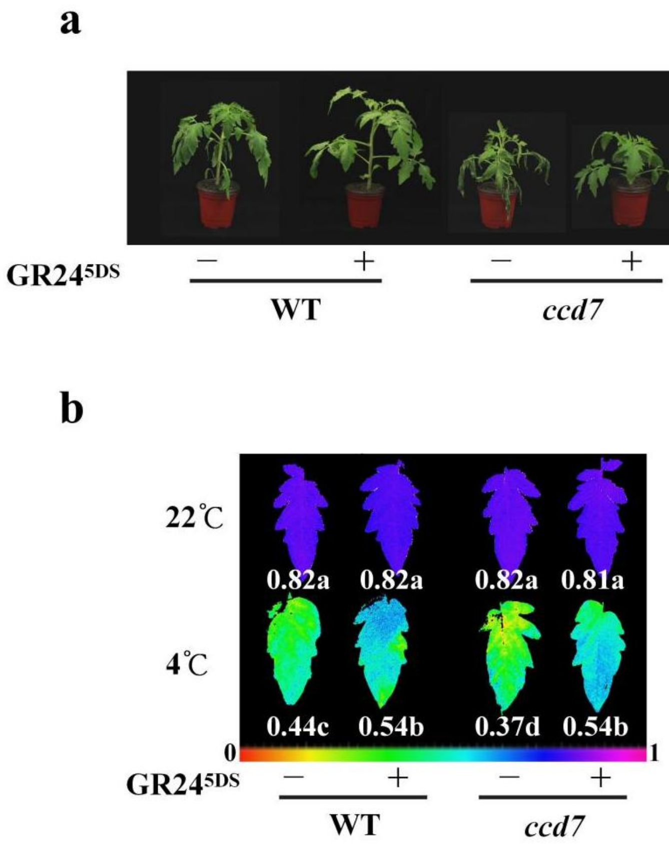

Fig. S10 Phenotypes of *ccd7* plants with or without GR24^{5DS} application after cold stress. a Phenotypes of plants after 7 d of cold stress at 4°C. **b** The maximum quantum yield of PSII (*Fv/Fm*) of the leaves from plants after 7 d of cold stress at 4°C. The GR24^{5DS} solution (3 μM, 15 mL) was applied to the roots of each plant 24 h before the cold stress. WT refers to the wild type (Condine Red), and *ccd7* refers to transgenic CRISPR-*ccd7* mutants. The plus and minus marks represent the application of GR24^{5DS} and water solution, respectively. For **b**, 15 leaves were used. Significant differences are indicated by different letters ($P < 0.05$, Tukey's test).

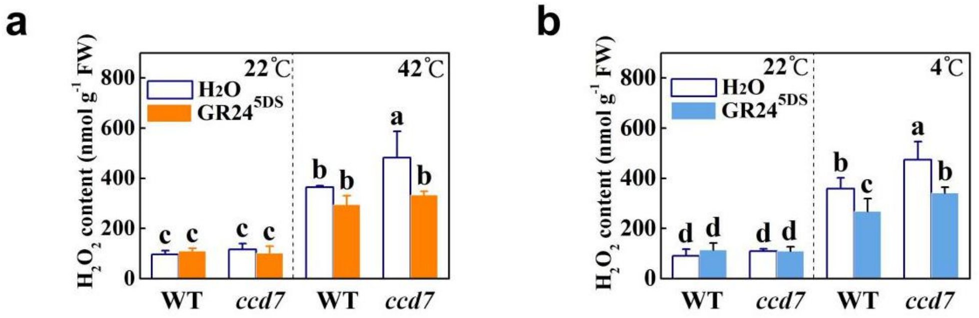

Fig. S11 Effects of *CCD7* mutation and GR24^{5DS} application on the accumulation of hydrogen peroxide in response to heat and cold stresses. a, **b** The hydrogen peroxide (H_2O_2) content in the leaves of plants under heat (**a**) or cold (**b**) stress. The GR24^{5DS} (3 μM, 15 mL) solution was applied to the roots of each plant 24 h before the heat or cold stress. WT refers to the wild type (Condine Red), and *ccd7* refers to transgenic CRISPR-*ccd7* mutants. Leaf tissues were collected after 48 h of heat stress at 42°C or 7 d of cold stress at 4°C. The results are the means ± SD of three biological replicates. Significant differences are indicated by different letters ($P < 0.05$, Tukey's test).

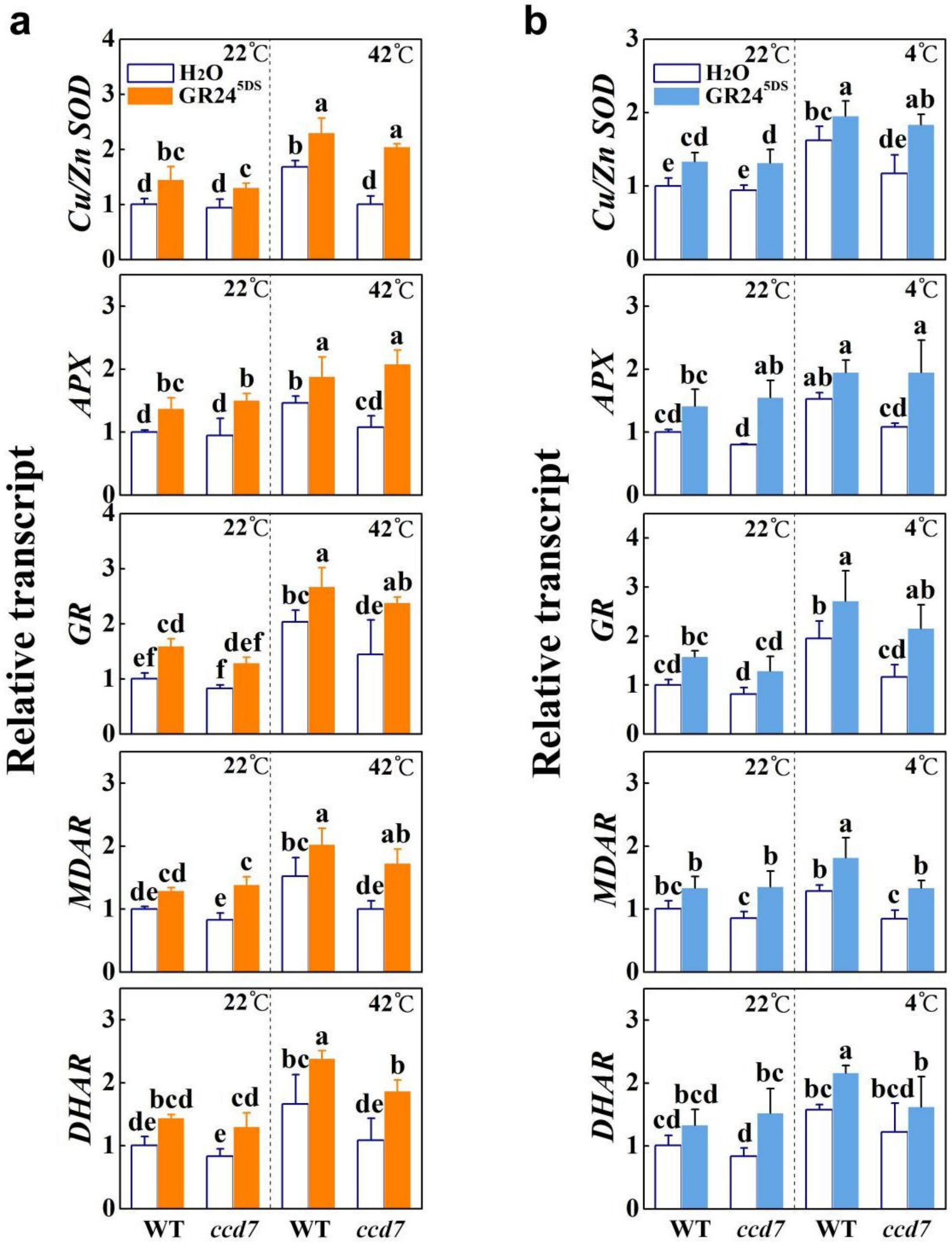

Fig. S12 Effects of *CCD7* mutation and GR24^{5DS} application on heat- and cold-induced antioxidant genes in tomato. Transcript levels of antioxidant-related genes in the leaves under hot conditions (**a**) or cold conditions (**b**). The GR24^{5DS} (3 μM, 15 mL) solution was applied to the roots of each plant 24 h before the cold or heat stress. WT refers to the wild type (Condine Red), and *ccd7* refers to transgenic CRISPR-*ccd7* mutants. Leaf tissues were collected after 12 h of heat stress at 42°C or cold stress at 4°C. The results are the means ± SD of three biological replicates. Significant differences are indicated by different letters ($P < 0.05$, Tukey's test).

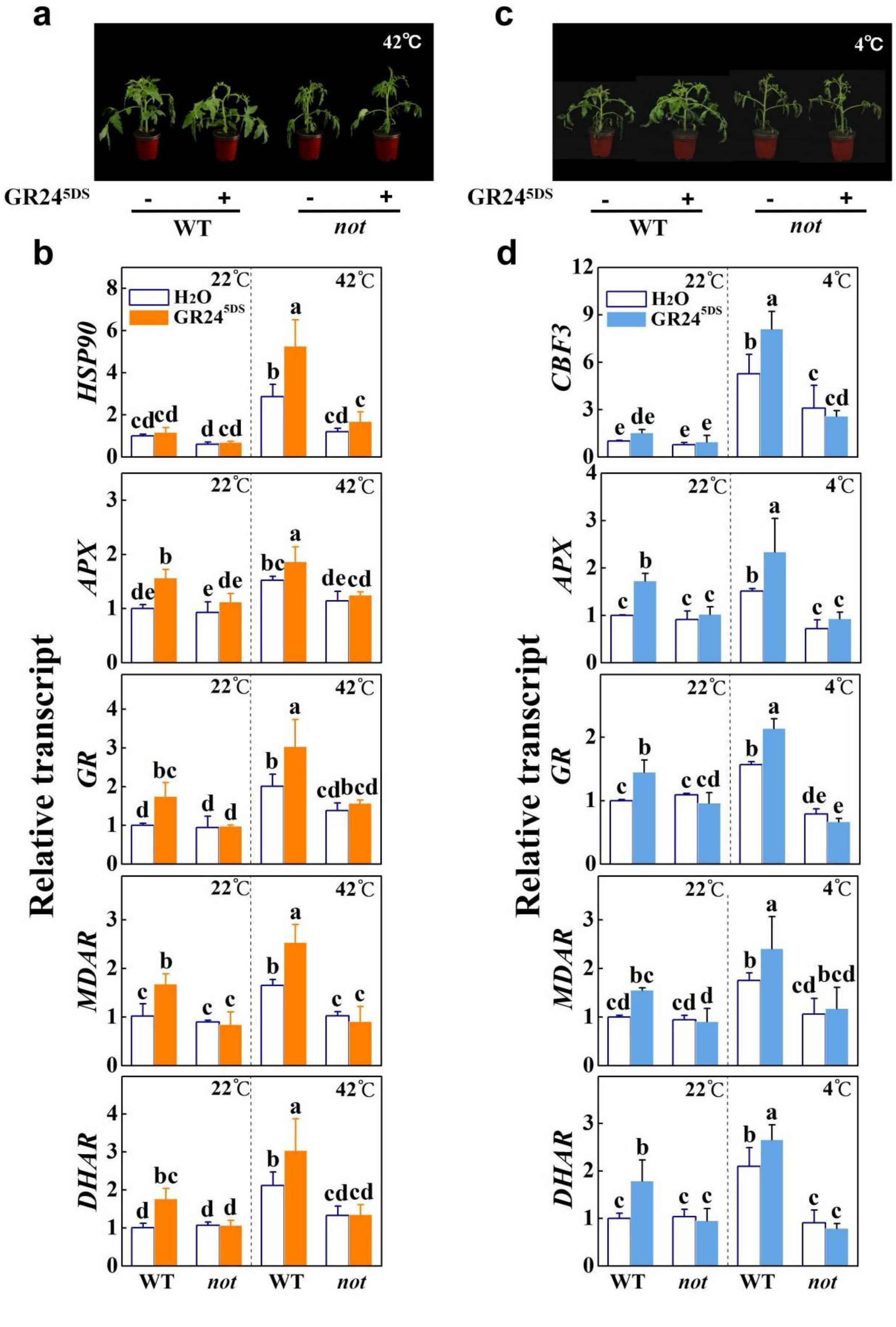

Fig. S13 Effects of GR24^{5DS} on heat- and cold-responsive and antioxidant genes in the ABA-deficient mutant *not*. a, **c** Phenotypes of plants after 48 h of heat stress at 42°C (**a**) or 7 d of cold stress at 4°C (**c**), respectively. **b**, **d** Transcript levels of genes in leaves after 12 h of heat stress at 42°C (**b**) or cold stress at 4°C (**d**). The GR24^{5DS} (3 μM, 15 mL) solution was applied to the roots of each plant 24 h before the heat or cold stress. WT refers to the wild type (Ailsa Craig). The plus

and minus marks represent the application of $GR24^{5DS}$ and water solution, respectively. The results are the means ± SD of three biological replicates. Significant differences are indicated by different letters ($P < 0.05$, Tukey's test).

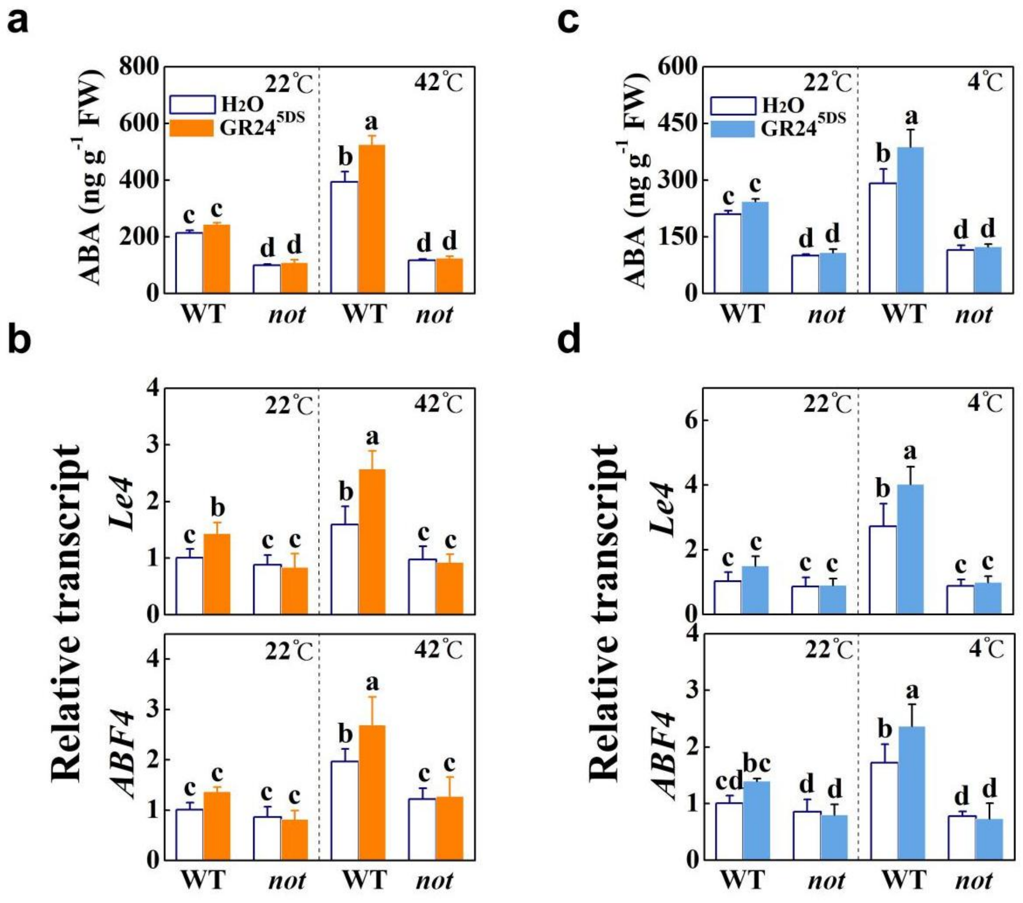

Fig. S14 Effects of $GR24^{5DS}$ on heat- and cold-triggered ABA biosynthesis and ABA-dependent transcriptional responses in the ABA-deficient mutant *not*. a, **c** ABA accumulation in the leaves of WT and *not* plants with or without $GR24^{5DS}$ treatment under heat and cold stresses. **b**, **d** Transcript levels of *Le4* and *ABF4* in the leaves of WT and *not* plants with or without $GR24^{5DS}$ application under heat and cold conditions. A $GR24^{5DS}$ (3 µM, 15 mL) solution was applied to the roots of each plant 24 h before stress. Leaf tissues were collected 12 h after the heat at 42°C or cold stress at 4°C. The results are the means ± SD of three biological replicates. Significant differences are indicated by different letters ($P < 0.05$, Tukey's test).

Table S1 PCR primers for VIGS vector construction.

Gene	Restriction sites		Forward primer	Reverse primer
CCD7	*Eco*RI	*Bam*HI	5'-CGgaattcCTAAAACAAAAGCCAAAATG-3'	5'-GCggatccATCCCAATAGGCAGTAAC-3'
CCD8	*Eco*RI	*Bam*HI	5'-CGgaattcTCTTCACATCTATGGCTTCT-3'	5'-GCggatccATCTGGTGGTGGAACTATTA-3'
MAX1	*Eco*RI	*Bam*HI	5'-CGgaattcGATTTGATCTCCTCCTTC-3'	5'-GCggatccGCATCAGCAACAATAACT-3'
MAX2	*Eco*RI	*Bam*HI	5'-CGgaattcTAGACCCACCATCATCAAT-3'	5'-GCggatccATGTCCCCAAGGAGAAAC-3'

Table S2 Primers used for qPCR assays.

Gene	Accession numbers	Forward primer	Reverse primer
ACTIN	Solyc11g005330	5'-TGTCCCTATTTACGAGGGTTATGC-3'	5'-CAGTTAAATCACGACCAGCAAGAT-3'
CCD7	Solyc01g090660	5'-AGCAGATTTTCCAGCGATGAA-3'	5'-AAAGGAAAATGGGGTAGCGC-3'
CCD8	Solyc08g066650	5'-CCTTCAACGGCAAGGATGTC-3'	5'-AAGGCCTCTTAGCACCACAA-3'
MAX1	Solyc08g062950	5'-GGTGTTACATTGTCTCGCCC-3'	5'-CTCCAAGAGCCAACCAAACC-3'
MAX2	Solyc12g010900	5'-TAAGACCAATGGAGGCGAGG-3'	5'-GGCCATTGTCTTCCATGCAA-3'
Cu/Zn-SOD	Solyc11g066390	5'-GGCCAATCTTTGACCCTTTA-3'	5'-AGTCCAGGAGCAAGTCCAGT-3'
APX	Solyc01g111510	5'-CGCCATATCACACAAGAAGC-3'	5'-TAACTCAGAGCCACCACTGC-3'
GR	Solyc09g091840	5'-GATGATGAAATGCGAGCTGT-3'	5'-TTGTGTTAGGGAGACGACCA-3'
MDAR	Solyc08g081530	5'-TCCGAACAAACATACCTGGA-3'	5'-CGTGTGTGCAGTTAGCAATG -3'
DHAR	Solyc05g054760	5'-CCCTGATGTCCTTGGAGACT-3'	5'-AAGAACCATTTGGGCTTGTC-3'
CBF1	Solyc03g026280	5'-GTGACTTCGTGGATGAGGAG-3'	5'-AGGCATCAGTTTCCACACAA-3'
CBF3	Solyc03g026270	5'-TGCCGGGTTTACTTACGAAT-3'	5'-TCAGCTTCCACATGATCTCC-3'
HSP70	Solyc04g011440	5'-TGCTGGAGGTGTTATGACCA-3'	5'-GACACCTCTTGGTGCTGGAG-3'
HSP90	Solyc12g015880	5'-CAAGCTTGGTATCCATGAGG-3'	5'-ATTCTGGCCTTCTTTCATCC-3'
NCED6	Solyc05g053530	5'-GGTCTTGAAGTTGTGGGTC-3'	5'-CGGCTGGTTTGAGTGA-3'
Le4	Solyc02g084850	5'-ACTCAAGGCATGGGTACTGG-3'	5'-CCTTCTTTCTCCTCCCACCT-3'
ABF4	Solyc11g044560	5'-GAAGAGTCTCAAGCGTTGT-3'	5'-TGATCCTCCAGTCCCAC-3'

The genes are identified from the Sol Genomics Network (http://solgenomics.net/)
